# The multifactorial nature of reproductive decision-making among women with multiple sclerosis

**DOI:** 10.18332/ejm/224169

**Published:** 2026-08-31

**Authors:** Panagiota Dourou, Kleanthi Gourounti, Vaidas Jotautis, Aikaterini Lykeridou, Sofia Theodorou, Anastasios Orologas, Aikaterini Sousamli, Nikolaos Petrogiannis, Antigoni Sarantaki

**Affiliations:** 1 Department of Midwifery, Faculty of Health and Care Sciences, University of West Attica, Athens, Greece; 2 Kauno Kolegija Higher Education Institution, Kaunas, Lithuania; 3 Department of Psychology, Greek Multiple Sclerosis Society, Athens, Greece; 4 Department of Neurology, Greek Multiple Sclerosis Society, Athens, Greece; 5 Athens Naval Hospital, Athens, Greece

**Keywords:** multiple sclerosis, reproductive decision-making, psychosocial adaptation, chronic illness adjustment, equity

## Abstract

**INTRODUCTION:**

Multiple sclerosis (MS) is a chronic neurological disease that frequently affects women during their reproductive years and may influence fertility intentions and family planning decisions. This study aimed to identify factors associated with reproductive choices among women with MS and to examine the role of psychological, social, and disease-related variables in fertility decision-making.

**METHODS:**

A cross-sectional study was conducted in Greece between August 2023 and December 2024, involving 103 women aged 18–50 years with neurologist-confirmed MS (mean age: 38.05 ± 7.01 years). Participants were recruited from the Naval Hospital of Athens and the Greek Multiple Sclerosis Society. Structured questionnaires assessed social support, psychological wellbeing, relationship dynamics, repro-ductive motivation, and quality of life. Associations between clinical, psychosocial, and demographic var-iables and childbearing were examined using univariate analyses and binary logistic regression.

**RESULTS:**

In univariate analyses, childbearing was associated with relapsing–remitting MS compared with progressive disease forms (73.9% vs 0%, p=0.001), marital status (all unmarried women were childless, p=0.001), fingolimod exposure (all users were childless, p=0.008), higher mental health scores (p=0.035), greater health-related distress (p=0.006), and stronger fatalistic motivation for parenthood (p=0.005). However, in multivariable logistic regression, none of these variables independently predicted childbearing status (all p>0.05).

**CONCLUSIONS:**

These findings suggest that reproductive decision-making among women with MS may involve multiple disease-related, psychological, and relationship-related considerations. Further studies with larger sample sizes are needed to confirm these associations.

## INTRODUCTION

Multiple sclerosis (MS) is a chronic neurological disorder that affects both physical and cognitive functions, with potential implications for fertility and pregnancy outcomes^1,2^. This condition is more prevalent in women than in men, particularly during their reproductive years, with onset typically occurring between the ages of 20 and 40 years^3^. Globally, about 2.8 million people are diagnosed with MS, with 1 million of these cases found in Europe^4^. In Greece, approximately 13000 individuals are affected by MS, with initial symptoms typically appearing between the third and fifth decades of life^5^.

The interplay between the disease and the side effects of medical treatments can profoundly impact reproductive health and decision-making in affected individuals^6,7^. Recent medical advancements have empowered many women with chronic illnesses and disabilities to conceive and embrace motherhood^8^. While research generally supports the safety of pregnancy for women with MS and indicates no detrimental effects on neonatal outcomes, there is evidence of a higher incidence of voluntary childlessness and pregnancy terminations among MS patients^9,10^. Considering that MS is often diagnosed during the reproductive years and primarily affects women, family planning decisions become a pivotal issue for this population^1,2,8^.

Globally, women with MS report significant concerns regarding pregnancy, including disease progression, treatment safety, and parenting capacity, which can influence reproductive decision-making^11-15^. To date, no peer-reviewed studies have specifically examined the determinants of fertility, reproductive decision-making, or childbearing patterns among women with MS in Greece. In cultural contexts where family remains a central social pillar, the psychological pressure to conform to traditional reproductive roles may conflict with the uncertainties associated with a chronic neurological diagnosis, making this population particularly relevant for examining the interplay between normative ‘fatalistic’ motivation and health-related quality of life^8^.

This study is grounded in a psychosocial framework drawing on elements of the Theory of Planned Behavior (TPB)^16^ and the Health Belief Model (HBM)^17^. Within this framework, subjective norms are reflected in relational and social contextual factors, while perceived behavioral control is represented by disease-related constraints and treatment considerations. In parallel, HBM-related constructs are captured through perceived health status, anticipated barriers to pregnancy, and psychological factors influencing reproductive decision-making. Women affected by chronic conditions such as MS represent a population that is both socially and medically vulnerable, with reproductive health needs that are frequently overlooked^8,9^. This study contributes valuable evidence aimed at enhancing health equity, patient-centered care, and informed decision-making for women with chronic neurological disorders.

Building on evidence that both disease-related and psychosocial factors shape fertility-related outcomes in women with MS, we hypothesized that clinical variables (such as disease progression and the use of disease-modifying therapies) and psychosocial characteristics (including mental health-related quality of life, marital status, and relationship quality) would be associated with childbearing status. Additionally, we examined the relationship between motivational, psychological, and relational factors, and both parity and attempts to conceive among childless women.

This study aimed to investigate the clinical, psychosocial, and relational determinants of reproductive decision-making among Greek women with MS.

## METHODS

### Study design and setting

This cross-sectional observational study was conducted in Athens, Greece, between 1 August 2023 and 31 December 2024, with participants recruited from the Medically Assisted Reproduction Unit (MARU), the Gynecology and Neurology Clinics of the Naval Hospital of Athens (NHA), and the Greek Multiple Sclerosis Society (GMSS).

### Inclusion and exclusion criteria

The study targeted women aged 18–50 years^18^ who had a neurologist-confirmed diagnosis of MS, verified through clinical examination and medical records. Participants were required to have been in a stable intimate relationship for at least one year and to have the cognitive capacity to understand the study procedures and independently complete the self-administered questionnaires. There were no restrictions on reproductive history, and women could be either childless or have biological children. All clinical forms of MS, including benign, relapsing–remitting, relapsing–progressive, and chronic–progressive, were eligible for inclusion.

Exclusion criteria included severe disability or advanced cognitive impairment that would impede the autonomous completion of the questionnaires, as well as major psychiatric disorders affecting cognition or decision-making capacity. Women undergoing antidepressant therapy at the time of enrollment were also excluded, in addition to those using medications known to impair fertility, such as hormonal contraceptives. The exclusion of participants receiving antidepressant medication was implemented to minimize pharmacological confounding of psychological, motivational, and quality-of-life outcomes, which were core variables in the study.

### Sampling procedure

Recruitment was purposefully diversified to capture both women currently seeking fertility assistance (MARU) and those in general neurological care. While this ensures a broad representation of reproductive experiences, it inherently weights the sample toward individuals with high fertility motivation and existing reproductive challenges.

A consecutive sampling approach was implemented, including all eligible women attending the Medically Assisted Reproduction Unit (MARU), as well as the Gynecology and Neurology Clinics of the Naval Hospital of Athens (NHA) and the Greek Multiple Sclerosis Society (GMSS) during the study period. Potential participants were identified during routine outpatient visits or support group meetings and were informed about the study by their treating clinician or a designated staff member. Those expressing interest were referred to the research team for eligibility screening. Women who met the inclusion criteria received a written information sheet and were invited to participate, and written informed consent was obtained prior to questionnaire administration. Participation was voluntary, with no use of peer recruitment or referral chains, and no financial or other incentives were provided.

### Sample size calculation

The required sample size was determined using G*Power 3.1 for a binary logistic regression analysis, assuming a medium effect size (OR=1.8), an alpha level of 0.05, and power of 0.80. The analysis indicated a minimum sample size of 92 participants.

### Data collection and measures

Data were collected using a structured self-administered questionnaire (Supplementary file Material 1), comprising demographic, clinical, psychosocial, and quality-of-life measures. The questionnaire included: 1) Demographics and reproductive history (age, education level, employment, marital status, number of children, pregnancies, fertility treatments); 2) Chronic disease history (MS duration, type, comorbidities, and medication use); 3) Oslo Social Support Scale (OSSS-3), assessing perceived social support across three dimensions (validated in Greek)^19,20^; 4) Dyadic Adjustment Scale (DAS), evaluating relationship quality (Greek version validated)^21,22^; 5) Center for Epidemiological Studies Depression Scale (CES-D), measuring depressive symptoms over the past week (Greek version validated)^23,24^; 5) Big Five Inventory (BFI), assessing five personality traits (Greek version validated)^25^; 6) Multiple Sclerosis Quality of Life-54 (MSQOL-54), evaluating quality of life across 12 domains and two composite scores (Greek version used)^26,27^; 7) Motivation for Parenthood Questionnaire (PM2), capturing four motivational dimensions (altruistic, fatalistic, narcissistic, and instrumental), validated in Greek through forward–backward translation, pilot testing, and psychometric evaluation (ICC=0.80, CFI=0.93, TLI=0.91, RMSEA=0.05, χ²/df=2.19)^28^; and 8) Positivity scale, an 8-item measure of positive orientation, validated in Greek (Cronbach’s α=0.86, ICC=0.82, CFI=0.94, TLI=0.92, RMSEA=0.048)^29^.

For all instruments, responses were recorded using standardized Likert-type scales, with higher scores indicating a greater presence of the measured construct unless otherwise specified. The OSSS-3 consists of three items (score range 3–14), with higher scores indicating stronger social support. The DAS includes 32 items (score range 0–151), with higher scores reflecting better relationship quality. The CES-D includes 20 items (score range 0–60), with higher scores indicating greater depressive symptomatology. The BFI comprises 44 items assessing five personality dimensions. The MSQOL-54 includes 54 items generating 12 subscale scores and two summary composite scores (physical and mental health), with higher scores reflecting better quality of life. The PM2 includes 20 items rated on a 5-point Likert scale, producing four motivational domains, with higher scores indicating stronger motivation. The Positivity Scale includes 8 items (score range 8–40), with higher scores reflecting a more positive outlook.

Questionnaires were completed either during clinical visits or at home and returned during subsequent appointments. Participants were provided with standardized instructions by the research team, emphasizing spontaneous and honest responses to ensure data validity.

### Study outcomes

Before data collection, the study outcomes were defined *a priori* to ensure methodological transparency and to minimize analytical bias. The primary outcome was the reproductive status of women with MS, operationalized as a binary variable indicating the presence or absence of children. The secondary outcomes included: 1) parity (total number of biological children); 2) attempt to conceive among childless participants during the study period; and 3) motivation for parenthood as assessed by the Parenthood Motivation Scale, which generates subscale scores across altruistic, fatalistic, narcissistic, and instrumental motives. These outcomes were selected based on theoretical relevance and prior literature on reproductive decision-making in chronic illness and were established before statistical analyses commenced^6,7,11-15^.

### Pilot testing and initial reliability procedures

Pilot testing was conducted to evaluate clarity, feasibility, and completion time of the study package in a small convenience subset of eligible women with MS who were not included in the final analytic sample. Participants were asked to comment on item wording, comprehension, and any ambiguities. Minor language and formatting adjustments were made to improve readability and reduce respondent burden without altering construct content. For instruments necessitating translation into Greek, such as the PM2 and the positivity scale, a forward–backward translation technique was utilized. Furthermore, the construct validity was evaluated through Exploratory and Confirmatory Factor Analyses (EFA/CFA) before undertaking the main analyses.

### Statistical analysis

Analyses were performed in SPSS v26. Continuous variables (e.g. age and scale/subscale scores) were summarized as means ± standard deviations (SDs), whereas categorical variables (e.g. marital status, MS course, treatment exposure) were summarized as frequencies and percentages. Normality of continuous variables was evaluated using the Kolmogorov–Smirnov test, supplemented by visual inspection of histograms and Q–Q plots. For between-group comparisons involving two independent groups, independent-samples t-tests were used for approximately normally distributed continuous outcomes; for comparisons involving three or more groups, one-way ANOVA was applied. Associations between categorical variables were examined using the chi-squared test, with Fisher’s exact test used when expected cell counts were small. Correlation analyses between continuous variables were performed using Pearson’s correlation coefficient (r).

Internal consistency reliability of scales was assessed using Cronbach’s α. For instruments undergoing validation procedures, factorial structure was examined using EFA/CFA, and test–retest reliability was estimated using the Intraclass Correlation Coefficient (ICC). To examine predictors of reproductive outcomes, binary logistic regression was conducted with childbearing status (yes, no) or attempted conception among childless women (yes, no) as dependent variables. Candidate predictors included demographic, clinical, psychosocial, and motivational variables that were theoretically relevant and/or significant in univariate analyses. For exploratory analyses of quality-of-life differences according to parity, age, and disease duration, adjusted linear regression models (ANCOVA-equivalent) were fitted for MSQOL-54 subscales that differed in univariate comparisons. Statistical significance was set at p<0.05. Multicollinearity among predictors (e.g. age, marital status, and MS course) was assessed using variance inflation factors (VIF) before regression analysis. A VIF >5 was considered indicative of moderate multicollinearity, while VIF >10 was interpreted as evidence of high multicollinearity, potentially compromising model stability^30^.

## RESULTS

### Participants’ characteristics

The study involved a population of 103 women, with a mean age of 38.05 years (SD=7.01) and with the majority being Greek (96.1%), married (79.4%), and having at least secondary or higher education. Most participants had at least one child (68.9%), with natural conception being the predominant method, although 29.6% had undergone *in vitro* fertilization. Clinically, the majority had relapsing–remitting MS (88.0%) and were receiving disease-modifying therapy, most commonly beta interferons (70.9%), while no participants reported comorbid chronic mental illness.

Women with children were significantly older than childless women (39.75 ± 5.7 vs 35.56 ± 8.7 years, p=0.017). In univariate analyses, childbearing was also associated with marital status, disease progression category, and treatment exposure. Most married women had children, whereas all unmarried women were childless. Women with relapsing–remitting MS were more frequently mothers than childless participants (73.9% vs 26.1%), while all women with chronic-progressive MS were childless (p=0.001). Beta interferon use was associated with a higher proportion of childbearing (78.1% vs 46.7% among non-users, p=0.003), whereas all women (n=4) receiving fingolimod were childless (p=0.008). No significant differences were observed between women with and without children in social support, dyadic adjustment, depressive symptoms, personality score, overall quality of life, physical health score, mental health score, positivity, education level, or years since MS diagnosis (Table 1).

**Table 1:** Association between childbearing status and demographic, clinical, and psychosocial variables among women with multiple sclerosis in Athens, Greece, August 2023–December 2024 (N=103)

*Variables*	*Having children*		*p*
	*No*	*Yes*	
	*Mean (SD)*		
Age (years) total study population	38.05 (7.01)		
Age (years)	35.56 (8.67)	39.75 (5.72)	**0.017**
OSSS-3	6.06 (1.50)	5.80 (1.22)	0.367
Dyadic Adjustment Scale (overall score)	67.54 (5.96)	68.38 (5.32)	0.485
CES-D score	20.58 (3.53)	20.14 (4.13)	0.608
BFI- Overall score	141.77 (9.62)	141.30 (9.82)	0.826
MSQOL-54-Overall Quality of Life	62.23 (16.79)	67.49 (10.91)	0.112
MSQOL-54-Physical Health Score	37.51 (13.36)	40.85 (7.98)	0.219
MSQOL-54-Mental Health Score	48.56 (16.76)	54.78 (12.56)	0.067
Positivity Scale score	23.59 (4.24)	22.46 (3.40)	0.154
	***n (%)***	***n (%)***	
**Educational level**			0.778
Middle school	1 (50)	1 (50)	
High school	7 (24.1)	22 (75.9)	
Vocational institute	3 (37.5)	5 (62.5)	
Technological Educational Institute	10 (34.5)	19 (65.5)	
Bachelor’s degree	9 (34.6)	17 (65.4)	
Master’s degree	1 (14.3)	6 (85.7)	
Doctoral degree	1 (50)	1 (50)	
**Marital status**			**0.001**
Married	13 (16)	68 (84)	
Unmarried	16 (100)	0 (0)	
Divorced	2 (50)	2 (50)	
Civil partnership	1 (100)	0 (0)	
**Years diagnosed with MS**			0.129
<1	3 (50)	3 (50)	
1	2 (40)	3 (60)	
2	7 (28)	18 (72)	
3	12 (24)	38 (76)	
>3	8 (51.7)	6 (42.9)	
**Disease progression category**			**0.001**
Benign	2 (50)	2 (50)	
Relapsing-remitting	23 (26.1)	65 (73.9)	
Relapsing-progressive	1 (50)	1 (50)	
Chronic-progressive	6 (100)	0 (0)	
**Beta interferons**			**0.003**
No	16 (53.3)	14 (46.7)	
Yes	16 (21.9)	57 (78.1)	
**Fingolimod**			**0.008**
No	28 (28.3)	71 (71.7)	
Yes	4 (100)	0 (0)	

MS: multiple sclerosis. BFI: Big Five Inventory. CES-D: Center for Epidemiological Studies – Depression. MSQOL: Multiple Sclerosis Quality of Life. OSSS-3: Oslo Social Support Scale -3. Continuous variables were compared using an independent-samples t-test as appropriate and categorical variables using chi-squared or Fisher’s exact test. Statistical significance was set at p<0.05.

In our research sample, approximately 30% of pregnancies were achieved through the use of assisted reproductive technologies. Additionally, infertility factors were identified in a substantial proportion of participants, including tubal (23.1%), male-factor (9.2%), ovarian (7.7%), and unexplained infertility (7.7%), which likely contributed to the increased use of assisted reproductive techniques, particularly *in vitro* fertilization (29.6%).

### Scales

Within our population, the OSSS-3 scores ranged from 4 to 10 (mean=5.89, SD=1.32), with a Cronbach’s alpha of 0.73, indicating acceptable reliability. Total DAS scores ranged from 54 to 96 (mean=68.12, SD= 5.51; α=0.70). Subscale scores and reliabilities were as follows (Dyadic consensus: 8–46 (mean=20.89, SD=5.12; α=0.80); Dyadic satisfaction: 23–37 (mean=28.40, SD=2.11; α=0.76); Dyadic cohesion: 7–20 (mean=15.26, SD=2.19; α=0.74) and affectional expression: 0–8 (mean=3.56, SD=1.33; α=0.70).

CES-D scores ranged from 12 to 32, with a mean of 20.27 (SD=3.95). The internal consistency of the scale was acceptable, with a Cronbach’s alpha of 0.73. The overall BFI scale demonstrated satisfactory reliability with a Cronbach’s alpha of 0.74. The subscales also showed good internal consistency: extraversion (α=0.76), agreeableness (α=0.77), conscientiousness (α=0.78), neuroticism (α=0.79), and openness (α=0.71).

With regard to the Multiple Sclerosis Quality of Life (MSQOL-54), descriptive statistics for each subscale showed acceptable to excellent internal consistency, with Cronbach’s alpha values ranging from 0.70 to 0.91. Physical function (α=0.87) had a mean score of 43.12 (SD=16.31), while the lowest mean was seen in role limitations due to physical problems (mean=13.24, SD=22.45; α=0.70). Cognitive function scored relatively high (mean=75.20, SD=17.38; α=0.73), as did social function (mean=61.89, SD=14.70; α=0.70). Emotional wellbeing, pain, energy, and sexual function domains showed moderate mean scores and satisfactory internal consistency. Individual item analysis revealed a mean of 40.93 (SD=15.25) for change in health and 49.30 (SD=22.29) for satisfaction with sexual function. The physical health composite score ranged from 11.96 to 66.01 (mean=39.83, SD=9.99), and the mental health composite score ranged from 13.30 to 88.86 (mean=52.83, SD=14.23).

Scores on the PM2 subscales were as follows: altruistic motivation ranged from 29 to 47 (mean=37.89, SD=4.31), fatalistic motivation from 40 to 53 (mean=46.19, SD=3.67), narcissistic motivation from 36 to 51 (mean=44.81, SD=3.25), and instrumental motivation from 46 to 57 (mean=51.73, SD=3.12), the last showing the highest average. Finally, scores on the positivity scale ranged from 15.00 to 36.00, with a mean of 22.82 (SD=3.71). The scale demonstrated satisfactory internal consistency (Cronbach’s α).

### Investigation of factors related to having children or attempting to have children

At the univariate level, multiple demographic, clinical, psychological, and relational variables were associated with childbearing and reproductive intentions. Tables 1–4 summarize these associations across parity status, attempts to conceive, treatment exposure, and motivational profiles.

**Table 2:** Association between parity (number of children) and demographic, clinical, and psychosocial variables among women with multiple sclerosis in Athens, Greece, August 2023–December 2024 (N=103)

*Variables*	*Number of children*			*p*
	*0*	*1*	*2*	
	*Mean (SD)*			
Age (years)	35.56 (8.67)	37.72 (5.02)	43.48 (5.1)	**0.001**
OSSS-3	6.06 (1.50)	5.70 (1.06)	6.00 (1.47)	0.457
Dyadic Adjustment Scale (overall score)	67.54 (5.96)	67.93 (6.11)	69.20 (3.39)	0.515
CES-D score	20.58 (3.53)	20.32 (4.15)	19.80 (4.16)	0.762
BFI- Overall score	141.77 (9.62)	141.95 (8.82)	140.12 (11.54)	0.735
MSQOL-54-Overall Quality of Life	62.23 (16.79)	65.91 (10.63)	70.33 (11.04)	0.070
MSQOL-54-Physical Health Score	37.51 (13.63)	39.64 (8.78)	43.10 (5.75)	0.132
MSQOL-54-Mental Health Score	48.56 (16.76)	53.97 (13.63)	56.23 (10.47)	0.100
Positivity Scale score	23.59 (4.24)	22.58 (3.23)	22.24 (3.76)	0.338
	***n (%)***	***n (%)***	***n (%)***	
**Education level**				0.079
Middle school	1 (50)	0 (0)	1 (50)	
High school	7 (24.1)	9 (31)	13 (44.8)	
Vocational institute	3 (37.5)	5 (62.5)	0 (0)	
Technological Educational Institute	10 (34.5)	12 (41.4)	7 (24.1)	
Bachelor’s degree	9 (34.6)	15 (57.7)	2 (7.7)	
Master’s degree	1 (14.3)	4 (57.1)	2 (28.6)	
Doctoral degree	1 (50)	1 (50)	0 (0)	
**Marital status**				**0.001**
Married	13 (16)	43 (53.1)	25 (30.9)	
Unmarried	16 (100)	0 (0)	0 (0)	
Divorced	2 (50)	2 (50)	0 (0)	
Civil partnership	1 (100)	0 (0)	0 (0)	
**Years diagnosed with MS**				**0.044**
<1	3 (50)	2 (33.3)	1 (16.7)	
1	2 (40)	3 (60)	0 (0)	
2	7 (28)	16 (64)	2 (8)	
3	12 (24)	21 (42)	17 (34)	
>3	8 (57.1)	3 (21.4)	3 (21.4)	
**Disease progression category**				**0.005**
Benign	2 (50)	1 (25)	1 (25)	
Relapsing-remitting	23 (26.1)	43 (48.9)	22 (25)	
Relapsing-progressive	1 (50)	1 (50)	0 (0)	
Chronic-progressive	6 (100)	0 (0)	0 (0)	
**Beta interferons**				**0.006**
No	16 (53.3)	11 (36.7)	3 (10)	
Yes	16 (21.9)	35 (47.9)	22 (30.1)	
**Fingolimod**				**0.011**
No	28 (28.3)	46 (46.5)	25 (25.3)	
Yes	4 (100)	0 (0)	0 (0)	

MS: multiple sclerosis. BFI: Big Five Inventory. CES-D: Center for Epidemiological Studies – Depression. MSQOL: Multiple Sclerosis Quality of Life. OSSS-3: Oslo Social Support Scale -3. Continuous variables were compared using an independent-samples t-test as appropriate and categorical variables using chi-squared or Fisher’s exact test. Statistical significance was set at p<0.05.

**Table 3:** Association between attempts to conceive among childless women with multiple sclerosis and demographic, clinical, and psychosocial variables in Athens, Greece, August 2023–December 2024 (N=32)

*Variables*	*Attempted conception*		*p*
	*No*	*Yes*	
	*Mean (SD)*		
Age (years)	35.16 (9.78)	36.15 (7.08)	0.756
OSSS-3	6.26 (1.52)	5.76 (1.48)	0.369
Dyadic Adjustment Scale (overall score)	68.63 (6.09)	65.83 (5.55)	0.200
CES-D score	20.52 (4.00)	20.66 (2.80)	0.916
BFI- Overall score	141.63 (10.86)	142.00 (7.69)	0.919
MSQOL-54-Overall Quality of Life	62.70 (12.23)	61.53 (22.44)	0.850
MSQOL-54-Physical Health Score	38.93 (13.39)	35.18 (13.62)	0.574
MSQOL-54-Mental Health Score	48.33 (13.76)	48.88 (21.02)	0.929
Positivity Scale score	23.89 (4.05)	23.15 (4.65)	0.636
	***n (%)***	***n (%)***	
**Education level**			0.702
Middle school	0 (0)	1 (100)	
High school	5 (71.4)	2 (28.6)	
Vocational institute	2 (66.7)	1 (33.3)	
Technological Educational Institute	5 (50)	5 (50)	
Bachelor’s degree	6 (66.7)	3 (33.3)	
Master’s degree	1 (100)	0 (0)	
Doctoral degree	0 (0)	1 (100)	
**Marital status**			**0.048**
Married	5 (38.5)	8 (61.5)	
Unmarried	13 (81.3)	3 (18.7)	
Divorced	1 (50)	1 (50)	
Civil partnership	0 (0)	1 (100)	
**Years diagnosed with MS**			0.510
<1	2 (66.7)	1 (33.3)	
1	0 (0)	2 (100)	
2	4 (57.1)	3 (42.9)	
3	7 (58.3)	5 (41.7)	
>3	6 (75)	2 (25)	
**Disease progression category**			0.122
Benign	0 (0)	2 (100)	
Relapsing-remitting	13 (56.5)	10 (43.5)	
Relapsing-progressive	1 (100)	0 (0)	
Chronic-progressive	5 (83.3)	1 (16.7)	
**Corticosteroids**			0.279
No	18 (64.3)	10 (35.7)	
Yes	1 (25)	3 (75)	
**Beta interferons**			0.719
No	10 (62.5)	6 (37.5)	
Yes	9 (56.3)	7 (43.8)	

MS: multiple sclerosis. BFI: Big Five Inventory. CES-D: Center for Epidemiological Studies – Depression. MSQOL: Multiple Sclerosis Quality of Life. OSSS-3: Oslo Social Support Scale -3. Continuous variables were compared using an independent-samples t-test as appropriate and categorical variables using chi-squared or Fisher’s exact test. Statistical significance was set at p<0.05.

**Table 4:** Multivariable logistic regression analyses examining factors associated with reproductive outcomes among women with multiple sclerosis in Athens, Greece, August 2023–December 2024

*Variables*	*AOR*	*95% CI*
**Childbearing among women with multiple sclerosis** (N=103)		
Age (years)	0.98	0.56–1.70
Marital status	0.11	0.01–1.42
Category of disease progression	0.29	0.01–10.08
Beta interferons	3.88	0.02–83.83
MSQOL-54 Emotional well-being	1.05	0.80–1.37
MSQOL-54 Health distress	1.06	0.77–1.46
PM2-Fatalistic	1.60	0.76–3.37
**Attempts to conceive among childless women with MS** (N=32)		
Affectional Expression Scale	0.43	0.18–0.99
Marital status	0.83	0.58–1.20

AOR: adjusted odds ratio. Binary logistic regression was performed. MS: multiple sclerosis. MSQOL: Multiple Sclerosis Quality of Life. Statistical significance was set at p<0.05.

*Factors associated with having children*

In the univariate analysis, age was significantly higher among women with children (mean=39.75, SD=5.72) compared to those without (mean=35.56, SD=8.67; p=0.017). Mental functioning (MSQOL-54) was significantly better in women with children (mean=56.74, SD=12.35) than those without (mean=50.87, SD=14.01; p=0.035). Health worries were also higher among mothers (mean=57.14, SD=16.93) compared to non-mothers (mean=44.06, SD=23.08; p=0.006). Fatalistic motivation was more pronounced in women with children (mean=47.86, SD=2.92) than in those without (mean=44.08, SD=3.50; p=0.005). Marital status showed a strong association (p=0.001); 84% of married women had children, while all unmarried women were childless. MS progression type was also significant (p=0.001); all women with chronic/progressive MS were childless, whereas 73.9% with relapsing/remitting MS had children. Beta interferon use was associated with childbearing (p=0.003); 78.1% of women receiving this treatment had children. Fingolimod use was negatively associated (p=0.008); all women on fingolimod (n=4) were childless.

In multivariable logistic regression analysis, no variable was independently associated with childbearing (all p>0.05). Similarly, treatment with beta interferons (OR=3.88; 95% CI: 0.018–83.827, p=0.621), emotional wellbeing (OR=1.048; 95% CI: 0.803–1.367, p=0.730), health distress (OR=1.059; 95% CI: 0.767–1.463, p=0.727), and fatalistic motivation (OR=1.597; 95% CI: 0.757–3.368, p=0.219) did not demonstrate independent associations with childbearing.

*Factors associated with the number of children*

In univariate analyses, parity was associated with age, selected MSQOL-54 domains, fatalistic motivation, marital status, disease duration, disease progression category, and treatment exposure (Table 2). Mean age increased across parity groups, from 35.56 ± 8.67 years among women without children to 37.72 ± 5.02 years among women with one child and 43.48 ± 5.10 years among women with two children (p=0.001)

(Table 2). Social functioning scores also increased with parity, with mean scores of 50.87 ± 14.01, 54.66 ± 12.12, and 60.48 ± 12.12 among women with no children, one child, and two children, respectively (p=0.021) (Supplementary file Table S1). Health perceptions were higher among women with two children compared with those with no children or one child (38.00 ± 18.92 vs 26.71 ± 12.08 and 28.66 ± 8.88, respectively; p=0.003) (Supplementary file Table S1). Health distress scores also differed across parity groups, increasing from 44.06 ± 23.08 among childless women to 55.11 ± 19.11 among women with one child and 60.80 ± 11.51 among women with two children (p=0.004) (Supplementary file Table S1). Fatalistic motivation differed significantly by parity status, with the highest mean score observed among women with one child (48.77 ± 2.48), compared with women without children (44.08 ± 3.50) and those with two children (46.50 ± 3.20; p=0.009) (Supplementary file Table S1).

Parity was also associated with marital status (p=0.001), as most married women had one or two children, whereas all unmarried women were childless (Table 2). Years since MS diagnosis differed across parity groups (p=0.044), with a higher proportion of childlessness observed among women diagnosed for more than three years (Table 2). Disease progression category was also associated with parity (p=0.005); all women with chronic-progressive MS were childless, whereas women with relapsing–remitting MS were more frequently represented among those with one or two children (Table 2). Treatment exposure showed similar univariate associations. Beta interferon use was associated with higher parity (p=0.006), while all women receiving fingolimod were childless (p=0.011 (Table 2). However, the fingolimod finding should be interpreted cautiously because only four participants were receiving this treatment.

Table 2 and Supplementary file Table S1 summarize the associations between parity and demographic, clinical, psychosocial, quality-of-life, and motivational variables.

Women without a history of pregnancy were significantly younger than those with ≥1 pregnancy (35.56 ± 8.67 vs 40.20 ± 6.01 years, p=0.017) and were more frequently receiving disease-modifying therapies (84.4% vs 56.3%, p=0.028). No significant differences were observed in disease course (p=0.91) or disability status (p=0.76) between the groups. Health perceptions were significantly higher among women with ≥1 pregnancy (31.31 ± 13.16 vs 26.71 ± 12.08, p=0.003), while mental health scores did not differ significantly (p=0.100) (Supplementary file Table S2).

Among parous women, those with ≥2 children were significantly older (43.48 ± 5.10 vs 37.72 ± 5.02 years, p=0.001) and reported higher health perceptions (38.00 ± 18.92 vs 28.66 ± 8.88, p=0.003), better social functioning (60.48 ± 12.12 vs 54.66 ± 12.12, p=0.021), and higher health distress scores (60.80 ± 11.51 vs 55.11 ± 19.11, p=0.004). Marital status (p=0.001), disease progression (p=0.005), and beta-interferon use (p=0.006) were also significantly associated with parity (Supplementary file Table S3).

In multivariable analyses, none of the examined variables remained independently associated with reproductive outcomes.

Considering that women with two or more pregnancies were significantly older than those with only one (43.5 vs 37.7 years), we employed age- and disease-duration–adjusted linear regression models (equivalent to ANCOVA) to ascertain whether parity remained independently associated with MSQOL-54 subscales. In unadjusted analyses, women with two or more pregnancies demonstrated more favorable scores in health perceptions, social function, and health distress. Upon adjusting for age and years since MS diagnosis, the association between parity and social function persisted as statistically significant (adjusted mean difference for two or more vs one pregnancy 6.2 points; 95% CI: 1.1 – 11.3, p=0.018) (data not shown).

Conversely, the unadjusted differences in health perceptions and health distress diminished post-adjustment and did not achieve statistical significance (health perceptions: adjusted mean difference 2.1; 95% CI: -2.9–7.1, p=0.41; health distress: adjusted mean difference -2.6; 95% CI: -7.8–2.6, p=0.32) (results not shown).

*Investigation of factors related to the attempt to have a child among childless women*

Women who have tried to conceive tend to score notably lower on the affectional expression scale compared to those who have not pursued such efforts (p=0.030). Furthermore, marital status is significantly correlated with attempts to conceive, with married women being more likely to pursue conception than their unmarried counterparts (p=0.048). Table 3 and Supplementary file Table S4 provide a summary of the association between the attempt to have a child among childless participants and various parameters.

*Multivariable analysis of factors associated with attempts to conceive among childless women*

The affectional expression scale has a statistically significant, negative, and independent association with the attempt to have children among childless participants (p=0.047, AOR=0.427; 95% CI: 0.184–0.990) (Table 4).

*Investigation of factors related to motivations for having children*

Altruistic motivation for parenthood was significantly associated with educational attainment (p=0.044). Time since MS diagnosis was significantly associated with altruistic motivation (p=0.040), with the highest scores observed in women diagnosed for 1 year (mean: 46.00 ± 1.41), compared to those diagnosed for <1 year (39.20 ± 3.27), 2 years (37.00 ± 3.74), 3 years (36.00 ± 4.34), and >3 years (37.80 ± 3.49). Table 5 provides a summary of the association between the factors of the Motivation for Parenthood Scale (PM2) and various categorical parameters.

**Table 5:** Association between Motivation for Parenthood (PM2) subscale scores and demographic and clinical characteristics among women with multiple sclerosis in Athens, Greece, August 2023–December 2024 (N=103)

*Factors of Motivation for Parenthood (PM2)*	*Subscale scores* *Mean (SD)*								*p*
	**Educational level**								
	Middle school	High school	Vocational training diploma	Technical university degree	Bachelor’s degree	Master’s degree	Doctoral degree		
Altruistic	33.00 (-)	38.20 (2.86)	34.00 (2.82)	36.16 (3.06)	40.37 (3.50)	34.66 (6.65)	43.50 (2.12)		**0.044**
Fatalistic	51.00 (-)	44.80 (1.64)	50.50 (0.70)	45.50 (4.27)	45.12 (3.04)	49.00 (4.58)	45.00 (5.65)		0.230
Narcissistic	45.00 (-)	46.20 (3.11)	41.50 (3.53)	46.33 (2.06)	44.25 (2.86)	45.00 (2.00)	42.00 (8.48)		0.444
Instrumental	51.00 (-)	51.40 (2.96)	54.50 (0.70)	52.50 (3.56)	51.14 (3.67)	52.00 (1.73)	49.50 (4.94)		0.808
	**Marital status**							
	Married		Unmarried		Divorced		Civil partnership		
Altruistic	36.62 (4.41)		40.40 (2.07)		38.50 (4.94)		45.00 (-)		0.211
Fatalistic	47.31 (3.28)		43.20 (3.56)		46.00 (4.24)		41.00 (-)		0.128
Narcissist	44.50 (3.07)		45.20 (2.38)		41.50 (7.77)		48.00 (-)		0.343
Instrumental	52.06 (3.06)		51.60 (2.88)		54.50 (2.12)		46 (-)		0.183
	**Years diagnosed with MS**								
	<1	1		2		3		>3	
Altruistic	39.20 (3.27)	46.00 (1.41)		37.00 (3.74)		36.00 (4.34)		37.80 (3.49)	0.040
Fatalistic	44.40 (3.78)	43.50 (3.53)		47.40 (2.88)		46.75 (3.95)		46.40 (4.15)	0.592
Narcissistic	45.80 (3.11)	44.50 (4.94)		43.80 (5.35)		44.75 (2.25)		44.80 (3.49)	0.939
Instrumental	51.40 (2.60)	46.00 (0.00)		52.40 (3.50)		52.87 (2.41)		52.50 (3.69)	0.083
	**Category of disease progression**								
	Benign		Relapsing/remitting		Relapsing/progressive		Chronic/progressive		
Altruistic	41.00 (1.41)		37.60 (4.64)		39.00 (4.24)		38.00 (-)		0.771
Fatalistic	42.50 (2.12)		46.30 (3.71)		46.50 (4.94)		48.00 (-)		0.542
Narcissistic	43.50 (0.70)		44.95 (3.44)		43.00 (5.65)		47.00 (-)		0.747
Instrumental	53.00 (2.82)		51.75 (3.27)		51.50 (4.94)		-		0.873
	**Corticosteroids**								
	No				Yes				
Altruistic	37.80 (3.89)				38.16 (5.98)				0.862
Fatalistic	46.42 (3.26)				45.33 (5.12)				0.530
Narcissist	44.61 (3.41)				45.50 (2.73)				0.568
	**Beta interferons**								
	No				Yes				
Altruistic	37.44 (3.98)				38.77 (5.01)				0.459
Fatalistic	46.22 (3.87)				46.11 (3.44)				0.943
Narcissistic	45.22 (2.60)				44.00 (4.33)				0.367
Instrumental	51.70 (3.19)				51.77 (3.15)				0.957
	**Glatiramer acetate**								
	No				Yes				
Altruistic	37.37 (4.10)				42.00 (4.35)				0.079
Fatalistic	46.33 (3.72)				45.00 (3.60)				0.563
Narcissistic	45.00 (3.34)				43.33 (2.08)				0.413
Instrumental	52.00 (2.89)				49.66 (4.72)				0.230

MS: multiple sclerosis. Group comparisons were performed using one-way analysis of variance (ANOVA) for categorical variables. Statistical significance was set at p<0.05.

### Correlations between the factors of the motivation for Parenthood Scale (PM2)

Correlation analyses demonstrated negative correlations between altruistic motivation and both fatalistic and instrumental motivation, with the strongest correlation observed between altruistic and fatalistic motivation. Narcissistic motivation was also negatively correlated with instrumental motivation (Supplementary file Table S5).

Regarding psychosocial variables, altruistic motivation showed moderate negative correlations with overall dyadic adjustment (r= -0.461, p=0.016), dyadic consensus (r= -0.435, p=0.023), and dyadic cohesion (r= -0.440, p=0.022). In contrast, fatalistic motivation demonstrated moderate positive correlations with overall dyadic adjustment (r=0.504, p=0.007), dyadic consensus (r=0.486, p=0.010), and dyadic cohesion (r=0.560, p=0.002) (Supplementary file Table S5).

Fatalistic motivation showed consistent moderate positive correlations with several MSQOL-54 domains, including physical functioning (r=0.412, p=0.033), energy (r=0.433, p=0.024), social functioning (r=0.465, p=0.019), cognitive functioning (r=0.528, p=0.005), health distress (r=0.422, p=0.028), physical health composite score (r=0.554, p=0.004), and mental health composite score (r=0.403, p=0.037) (Supplementary file Table S5).

## DISCUSSION

This study examined clinical, psychosocial, and relational factors associated with reproductive outcomes among women with MS. Women with children were older and reported higher mental functioning, greater health-related concerns, and stronger fatalistic motivation for parenthood. Childbearing was also associated with marital status and disease course, with higher proportions of mothers among married women and those with relapsing–remitting MS. However, these associations should be interpreted cautiously given the cross-sectional design and the recruitment of a medically engaged cohort, including women attending a medically assisted reproduction unit.

A key finding was that although several variables were associated with childbearing in univariate analyses, none remained independently significant in the multivariable regression model. This suggests that reproductive decision-making among women with MS is shaped by complex and interdependent influences, including disease characteristics, treatment considerations, relationship context, psychological functioning, and health-related concerns^11,31-34^. These findings emphasize that reproductive choices cannot be explained by single predictors but instead reflect multifactorial decision processes^11,35^. Clinically, this highlights the importance of individualized counseling that integrates medical, psychosocial, and relational dimensions of reproductive decision-making^9,36^.

One notable finding was the positive association between fatalistic motivation for parenthood and higher mental health-related quality of life. Although the concept of fatalism is often associated with passivity or perceived lack of control, in the context of reproductive decision-making in chronic illness, it may represent acceptance of normative life transitions^8,12^. In this cohort, fatalistic motivation appeared to reflect an adaptive cognitive orientation in which parenthood is perceived as a meaningful life event rather than a decision requiring prolonged deliberation. This interpretation is supported by its association with higher levels of mental health, cognitive functioning, energy, and social functioning. Viewing parenthood as part of a natural life trajectory may reduce anxiety regarding disease uncertainty and contribute to psychological stability^5,8^.

Importantly, this form of fatalistic motivation appears distinct from maladaptive fatalism characterized by helplessness or hopelessness. Participants with stronger fatalistic motivation reported better psychosocial functioning and higher perceived quality of life, suggesting that such beliefs may coexist with active coping strategies.

The proportion of abortions observed in this cohort was approximately 19%, which is consistent with national estimates in Greece, indicating that induced abortions occur in roughly 20–25% of pregnancies among women of reproductive age^36^. Although methodological differences limit direct comparisons, the findings do not suggest an increased abortion rate among women with MS.

Treatment-related findings also revealed important patterns. Women receiving beta interferons were more likely to have children, whereas fingolimod use was strongly associated with childlessness in this sample. Interpretation of this finding requires caution due to the small number of fingolimod users. Fingolimod is a high-efficacy disease-modifying therapy with known teratogenic risks and requires strict contraception during treatment and a washout period prior to conception^9,10^. Consequently, clinicians often recommend postponing pregnancy while receiving this therapy. Additionally, fingolimod is typically prescribed to patients with higher disease activity, which may itself influence reproductive decisions due to concerns about disease progression and future parenting capacity^9^.

Given the cross-sectional design of the study, it is not possible to determine whether fingolimod exposure preceded decisions regarding childbearing or whether treatment selection occurred after reproductive plans were established. Therefore, the association likely reflects intertwined clinical and psychosocial processes rather than a direct causal relationship.

Among childless women, those who had attempted to conceive reported lower levels of affectional expression within their relationships and were more frequently married. Higher level of education was associated with stronger altruistic parenting motivations, while a more recent MS diagnosis was linked with increased altruistic motivation. Additionally, fatalistic motivation demonstrated a positive correlation with quality-of-life measures.

These results align with previous research indicating that concerns related to disease progression, treatment safety, and parenting ability influence reproductive decision-making among women with MS^31^. Previous studies have reported higher rates of voluntary childlessness among women with MS due to fears regarding disability progression, treatment risks, and potential impacts on child wellbeing^33-35^. For example, European survey data indicate that more than half of women with MS report that the disease influences their family planning decisions, while a substantial proportion decide against having children due to MS-related concerns^36^.

In the present study, higher health-related distress was observed among mothers, suggesting that concerns regarding symptoms, parenting capacity, and long-term health persist even after childbirth. At the same time, mothers reported better mental functioning and stronger fatalistic motivation, indicating that adaptive cognitive framing may coexist with ongoing health-related concerns.

While our findings indicate that no variables remained independently associated with childbearing in multivariable analysis, previous studies have reported associations between reproductive outcomes and factors such as disease severity, treatment exposure, and sociodemographic characteristics^11,31,33^. However, these associations have not been consistently observed across studies. The differences between our findings and prior literature may be explained by variations in study design, sample size, and population characteristics, including the recruitment of participants from fertility-focused settings and the inclusion of women in stable relationships in the present study. Additionally, the relatively small sample size and potential interrelationships among clinical and psychosocial variables may have reduced the ability to detect independent effects in multivariable models.

### Strengths and limitations

The study has several strengths. It contributes to an underexplored area of reproductive decision-making in women with MS and includes validation of the PM2 and positivity scale in the Greek population using robust psychometric methods, including exploratory and confirmatory factor analysis. The use of multidimensional measures allowed a comprehensive assessment of psychological, relational, and clinical factors associated with reproductive choices. However, several limitations should be considered. The cross-sectional design prevents causal interpretation of observed associations. Reverse causality cannot be excluded, as increased health-related concerns among mothers may reflect parenting responsibilities rather than factors preceding childbearing decisions. Treatment- and disease-related associations may also reflect underlying disease severity or prior reproductive choices rather than direct treatment effects. The sampling strategy should also be considered when interpreting the findings. A consecutive sampling approach was employed across multiple recruitment sites, including a Medically Assisted Reproduction Unit (MARU), hospital-based neurology and gynecology clinics, and a patient organization. While this strategy allowed for the inclusion of women with diverse clinical and reproductive experiences, it may have introduced selection bias, particularly due to the inclusion of participants from a fertility care setting. Women accessing assisted reproduction services are more likely to have higher fertility motivation, greater awareness of reproductive options, and potentially more complex fertility challenges compared to the general MS population. This is reflected in the relatively high proportion of pregnancies achieved through assisted reproductive techniques (approximately 30%), which may influence the observed associations between clinical and psychosocial variables and childbearing outcomes. Consequently, the findings may be more representative of women with MS actively engaged in fertility planning rather than the broader population of reproductive-age women with MS. Additionally, the inclusion criterion requiring participants to be in a stable relationship for at least one year further limits generalizability. Given the strong association between marital status and childbearing observed in this study, the results should be interpreted within the context of a relationship-enriched sample. Future research should aim to include single women and individuals with diverse relationship statuses to better capture the full spectrum of reproductive decision-making in MS. Furthermore, residual confounding cannot be excluded, as potentially relevant variables – such as socioeconomic status, access to fertility services, partner characteristics, cultural or religious beliefs, and physician counseling practices – were not explicitly controlled for in the analysis. Given the number of statistical comparisons performed across multiple clinical, psychological, and relational variables, there is also a risk of type I error, whereby some statistically significant findings may have occurred by chance. In addition, the use of self-reported data introduces the possibility of recall bias and social desirability bias, particularly in sensitive domains such as reproductive intentions, relationship quality, and psychological well-being. Additional limitations include the absence of partner perspectives, which may represent a key determinant of reproductive decision-making in chronic illness contexts. Furthermore, obstetric and neonatal outcomes were not examined in detail. Some psychometric findings also warrant cautious interpretation; for example, the OSSS-3 demonstrated potential multidimensionality, and certain items within the dyadic adjustment scale and CES-D showed lower internal consistency, possibly reflecting overlap between depressive symptoms and MS-related somatic features. Another limitation is the exclusion of women receiving antidepressant medication. While this was intended to reduce pharmacological confounding of psychological, motivational, and quality-of-life outcomes, it may limit the generalizability of the findings to women with MS and comorbid depression or treated mood symptoms.

### Future studies

Future studies should include these women and account for depressive symptom severity and psychotropic medication use in adjusted analyses. Overall, reproductive decision-making among a female population with MS appears to be shaped by complex interactions between disease characteristics, treatment considerations, psychological adaptation, and relationship context. Future studies should use longitudinal and adequately powered multicenter designs to clarify the temporal relationships between MS course, treatment trajectories, psychosocial factors, and reproductive decisions. Further research should also examine therapy-specific family planning pathways, the role of medically assisted reproduction, and partner perspectives, ideally using mixed-methods approaches to better capture the clinical, relational, and cultural dimensions of reproductive decision-making.

## CONCLUSIONS

This study identified several factors associated with childbearing in women with MS, including age, marital status, disease progression, and psychosocial characteristics. Women with relapsing–remitting MS and those receiving beta interferons were more likely to have children in univariate analyses; however, no variables remained independently associated with childbearing in the multivariable model. Higher mental health scores, fatalistic motivation, and more positive health perceptions were observed among women with children, alongside greater health-related distress. Given the cross-sectional design, these findings should be interpreted with caution, and further longitudinal research is warranted.

## Supplementary Material



## Data Availability

The data supporting this research are available from the authors on reasonable request.
