# Supplementary material for: The multifactorial nature of reproductive decision-making among women with multiple sclerosis
Source: Eur J Midwifery. 2026 Aug 31;10:10.18332/ejm/224169. doi: 10.18332/ejm/224169 (PMC13531883; doi:10.18332/ejm/224169)
Supplement: Supplementary file 1 [file EJM-10-38-s1.pdf]

EUROPEAN JOURNAL
OF MIDWIFERY

Supplementary file

© 2026 Dourou P. et al.

DOI:
10.18332/ejm/224169

The content has been provided by the author(s) and has not been reviewed, verified, or endorsed by European Publishing. It may not have undergone peer review. The views, opinions, and recommendations expressed are solely those of the author(s) and do not necessarily reflect the position of European Publishing. European Publishing accepts no responsibility or liability for any consequences arising from the use of, or reliance on, this content.

ΔΗΜΟΓΡΑΦΙΚΑ ΣΤΟΙΧΕΙΑ

Ημερομηνία Συμπλήρωσης:…………………………..

Ηλικία : …………………………………………………………………..

Εθνικότητα:

☐ Ελληνική
☐ Άλλη

Αν έχετε άλλη εθνικότητα ποια είναι:……………………………………

Μορφωτικό επίπεδο:

☐ Δημοτικό
☐ Γυμνάσιο
☐ Λύκειο
☐ ΙΕΚ
☐ ΤΕΙ
☐ ΑΕΙ
☐ Μεταπτυχιακό
☐ Διδακτορικό

Επάγγελμα: ……………………………………………………………

Οικογενειακή κατάσταση:

☐ Έγγαμη
☐ Άγαμη
☐ Διαζευγμένη
☐ Χήρα
☐ Σύμφωνο Συμβίωσης
☐ Σε σχέση

ΜΑΙΕΥΤΙΚΟ-ΓΥΝΑΙΚΟΛΟΓΙΚΟ ΙΣΤΟΡΙΚΟ

Αριθμός παιδιών:

0
1
2
3
>3

Εάν έχετε παιδιά, με ποιον από τους παρακάτω τρόπους αποκτήσατε παιδί:

Φυσική σύλληψη
Εξωσωματική Γονιμοποίηση Σπερματέγχυση
Υιοθεσία
Άλλο:………………………………………………………………..

Με ποιον τρόπο γεννήσατε:

Δεν γέννησα
Φυσιολογικός τοκετός ≥37 εβδομάδων κύηση με επισκληρίδιο αναισθησία
Φυσιολογικός τοκετός <37 εβδομάδων κύηση με επισκληρίδιο αναισθησία Φυσιολογικός τοκετός ≥37 εβδομάδων κύηση χωρίς επισκληρίδιο αναισθησία Φυσιολογικός τοκετός <37 εβδομάδων κύηση χωρίς επισκληρίδιο αναισθησία Καισαρική τομή ≥37 εβδομάδων κύηση
Καισαρική τομή <37 εβδομάδων κύηση

Εάν δεν έχετε παιδιά, έχετε κάνει προσπάθεια για παιδί:

Ναι Όχι

Παράγοντας Υπογονιμότητας:

Κανένας
Σαλπιγγικός
Ωοθηκικός
Ηλικία
Ενδομητρίωση
Ανδρικός

Ανεξήγητος
Άλλο :…………………………………………………………………………..

Αριθμός αποβολών:

- 0
- 1
- 2
- 3
- >3

Αριθμός τεχνητών εκτρώσεων:

- 0
- 1
- 2
- 3
- >3

Γυναικολογικό χειρουργεία:

………………………………………………………………………………

ΙΣΤΟΡΙΚΟ ΧΡΟΝΙΑΣ ΝΟΣΟΥ

Πάσχετε από κάποια χρόνια ψυχική νόσο :

Όχι
Ναι Εάν ναι ποια:…………………………………………………

Πάσχετε από κάποια χρόνια σωματική νόσο :

Όχι
Ναι Εάν ναι ποια:…………………………………………………………

Πόσα χρόνια έχετε διεγνωσθεί με Σκλήρυνση κατά Πλάκας:

- <1
- 1
- 2
- 3
- >3

Ανάλογα με την εξέλιξη της νόσου, σε ποια κατηγορία ανήκετε:

- Καλοήθης
- Υποτροπιάζουσα / Διαλείπουσα
- Υποτροπιάζουσα / Επιδεινούμενη
- Χρονία / επιδεινούμενη

Τι θεραπεία λαμβάνετε:

- Κορτικοειδή
- Ιντερφερόνες β
- Οξική γλατιραμέρη
- Ναταλιζουμάμπη
- Φινγκολιμόδη
- Μιτοξανδρόνη
- Άλλο:………………………………………………………………………………

Καθε πότε λαμβάνετε την θεραπεία:

- Καθημερινά
- Μία φορά τον μήνα

Άλλο:…………………………………………………....................................

Oslo Social Support Scale (OSSS-3)

Oslo 1: Πόσα άτομα βρίσκονται κοντά σας, ώστε να μπορείτε να στηρίζεστε σε εκείνα όποτε αντιμετωπίζετε σοβαρά προσωπικά προβλήματα;

- ☐ Κανένα άτομο
- ☐ 1 - 2 άτομα
- ☐ 3-5 άτομα
- ☐ 6 ή παραπάνω άτομα

Oslo 2: Πόσο ενδιαφέρον δείχνουν τα άτομα σε αυτά που κάνετε;

- ☐ Πολύ ενδιαφέρον
- ☐ Δείχνουν κάποιο ενδιαφέρον
- ☐ Δεν είμαι σίγουρη
- ☐ Δείχνουν λίγο ενδιαφέρον
- ☐ Δε δείχνουν ενδιαφέρον

Oslo 3: Πόσο εύκολα λαμβάνετε πρακτική βοήθεια όταν τη χρειάζεστε από τους κοντινούς σας ανθρώπους;

- ☐ Πολύ εύκολα
- ☐ Εύκολα
- ☐ Υπάρχουν πιθανότητες
- ☐ Δύσκολα
- ☐ Πολύ δύσκολα

DYADIC ADJUSTMENT SCALE

Τα περισσότερα ζευγάρια έχουν διαφωνίες στις μεταξύ τους σχέσεις. Σας παρακαλώ πολύ να διαβάσετε προσεκτικά τις παρακάτω δηλώσεις και να επισημάνετε το βαθμό στον οποίο συμφωνείτε ή διαφωνείτε με το/τη σύντροφό σας σε κάθε μία από αυτές. (Βάλτε ένα √ για να δηλώσετε την απάντηση που ταιριάζει καλύτερα σε σας).

	Συμφωνώ πάντα	Συμφωνώ σχεδόν πάντα	Άλλοτε συμφωνώ και άλλοτε διαφωνώ	Διαφωνώ συχνά	Διαφωνώ σχεδόν πάντα	Διαφωνώ πάντα
1. Διαχείριση οικονομικών						
2. Θέματα διασκέδασης						
3. Θρησκευτικά ζητήματα						
4. Έκφραση στοργής						

	Συμφωνώ πάντα	Συμφωνώ σχεδόν πάντα	Άλλοτε συμφωνώ και άλλοτε διαφωνώ	Διαφωνώ συχνά	Διαφωνώ σχεδόν πάντα	Διαφωνώ πάντα
5. Φίλοι						
6. Σεξουαλικές σχέσεις						
7. Ορθότητα πράξεων						

8. Φιλοσοφία ζωής						
9. Τρόποι αντιμετώπισης γονέων ή πεθερικών						
10. Σκοποί, στόχοι και πράγματα που θεωρούνται σημαντικά						
11. Χρόνος που περνάτε μαζί						
12. Λήψη σημαντικών αποφάσεων						
13. Ασχολίες νοικοκυριού						
14. Χόμπι και δραστηριότητες διασκέδασης						
15. Αποφάσεις για την επαγγελματική σταδιοδρομία						

	Πάντα	Τις περισσότερες φορές	Συχνά	Περιστασιακά	Σπάνια	Ποτέ
16. Πόσο συχνά συζητάτε ή έχετε σκεφτεί για διαζύγιο, χωρισμό ή λήξη της σχέσης σας;						

17. Πόσο συχνά εσείς ή ο/η σύντροφός σας εγκαταλείπετε το σπίτι μετά από καυγά;						
18. Σε γενικές γραμμές, πόσο συχνά σκέφτεστε ότι τα πράγματα ανάμεσα σ' εσάς και τον/τη σύντροφό σας πηγαίνουν καλά;						
19. Έχετε εμπιστοσύνη στον/στην σύντροφό σας;						
20. Έχετε μετανιώσει ποτέ που παντρευτήκατε (ή συζείτε);						
21. Πόσο συχνά καυγαδίζετε με τον/την σύντροφό σας;						
22. Πόσο συχνά «εκνευρίζετε ο ένας τον άλλο»;						

	Κάθε μέρα	Σχεδόν κάθε μέρα	Περιστασιακά	Σπάνια	Ποτέ
23. Φιλάτε το/τη σύντροφό σας;					

	Σε όλες	Στις περισσότερες	Σε μερικές	Σε ελάχιστες	Σε καμία
24. Συμμετέχετε μαζί με τον/την σύντροφό σας σε δραστηριό-τητες ή ενδιαφέρο-ντα εκτός σπιτιού;					

Πόσο συχνά θα λέγατε ότι τα ακόλουθα γεγονότα συμβαίνουν με τον/την σύντροφό σας;

	Ναι	Όχι
29. Ήσασταν πολύ κουρασμένοι για σεξουαλική επαφή		

30. Δεν δείχνατε έμπρακτα την αγάπη σας		

	Ποτέ	Λιγότερο από 1 φορά το μήνα	1 ή 2 φορές το μήνα	1 ή 2 φορές την εβδομάδα	1 φορά την ημέρα	Συχνότερα
25. Έχετε μια ενδιαφέρουσα ανταλλαγή ιδεών και απόψεων						
26. Χαίρεστε και γελάτε μαζί						
27. Συζητάτε ήρεμα κάτι						
28. Συνεργάζεστε σε μια εργασία						

Υπάρχουν κάποια ζητήματα στα οποία τα ζευγάρια κάποιες φορές συμφωνούν και κάποιες άλλες διαφωνούν. Αναφέρετε εάν κάποιες από τις παρακάτω δηλώσεις προκάλεσαν διαφωνία απόψεων ή ήταν προβλήματα για τη σχέση σας κατά τη διάρκεια των προηγούμενων εβδομάδων (σημειώστε ναι ή όχι).

31. Οι λέξεις στην παρακάτω γραμμή αναφέρονται σε διαφορετικό βαθμό ικανοποίησης από τη σχέση σας. Η λέξη «ευχαριστημένος» αναφέρεται στο βαθμό ικανοποίησης των περισσότερων ανθρώπων από τη σχέση τους. Παρακαλώ <u>κυκλώστε</u> τον αστερίσκο που περιγράφει καλύτερα το δικό σας βαθμό ικανοποίησης από τη σχέση σας, έχοντας στο νου σας οτιδήποτε αφορά τη σχέση σας.

*	*	*	*	*	*	*
Εξαιρετικά δυσαρεστη-μένη	Αρκετά δυσαρεστη-μένη	Λίγο δυσαρεστη-μένη	Ευχαριστη-μένη	Πολύ ευχαριστη-μένη	Εξαιρετικά ευχαριστη-μένη	Τέλεια

32. Βάλτε ένα √ σε εκείνη την πρόταση από τις ακόλουθες που περιγράφει καλύτερα το πως αισθάνεστε για το μέλλον της σχέσης σας.

	Θέλω πάση θυσία να πετύχει η σχέση μου, και θα έκανα τα αδύνατα δυνατά για να πετύχει
	Θέλω πάρα πολύ να πετύχει η σχέση μου, και θα κάνω ότι μπορώ για να πετύχει
	Θέλω πάρα πολύ να πετύχει η σχέση μου, και θα κάνω αυτό που πρέπει από την πλευρά μου για να πετύχει
	Θα ήταν όμορφο αν πετύχαινε η σχέση μου, αλλά δεν μπορώ να κάνω τίποτα περισσότερο από όσα έχω κάνει μέχρι τώρα για να τη βοηθήσω να πετύχει
	Θα ήταν όμορφο αν πετύχαινε η σχέση μου, αλλά αρνούμαι να κάνω κάτι περισσότερο από ότι έχω κάνει μέχρι τώρα για να την διατηρήσω
	Η σχέση μου δεν μπορεί να είναι ποτέ επιτυχημένη, και δεν μπορώ να κάνω τίποτα άλλο για να την συνεχίσω

ΚΛΙΜΑΚΑ CENTER FOR EPIDEMIOLOGICAL STUDIES - DEPRESSION SCALE (CES - D)

Παρακάτω είναι μια σειρά από καταστάσεις που πιθανόν να περάσατε ή να αισθανθήκατε. Σας παρακαλώ σημειώστε μου πόσο συχνά αισθανθήκατε έτσι τις 7 τελευταίες μέρες.

	**α**	**β**	**γ**	**δ**
	Σπάνια ή καθόλου (λιγ. από 1 μέρα)	Μερικές φορές (1-2 μέρες)	Αρκετές φορές (3-4 μέρες)	Τον περισσότερο καιρό (5-7 μέρες)
1. Σας ενοχλούν πράγματα που συνήθως δεν σας ενοχλούν				
2. Η όρεξη σας είναι κακή ώστε να μη θέλετε να τρώτε				
3. Νιώθατε ότι δεν μπορούσατε να απαλλαγείτε από την κακοκεφιά αμέσως, ακόμα κι αν οι φίλοι ή η οικογένεια σας, σας βοηθούσαν				
4. Νιώθετε ότι είσαστε εξίσου ικανός με τους άλλους				
5. Έχετε δυσκολία να συγκεντρωθείτε σ' αυτό που κάνατε				
6. Νιώθετε μελαγχολική				
7. Νιώθετε πως ότι κάνετε το κάνετε με μεγάλη προσπάθεια, πίεση				
8. Νιώθετε ελπίδα για το μέλλον				
9. Σκεφτόσαστε ότι η μέχρι τώρα ζωή σας ήταν μια αποτυχία				
10. Νιώθετε φοβισμένη				

11.Εχετε ανήσυχο ύπνο				
12. Είσαστε ευτυχισμένη				
13. Μιλάτε λιγότερο από το συνηθισμένο				
14. Νιώθετε μοναξιά				
15. Πιστεύετε ότι οι άνθρωποι δεν είναι φιλικοί μαζί σας				
16. Ευχαριστιέστε (χαιρόσαστε τη ζωή)				
17. Κλαίτε με λυγμούς				
18. Νιώθετε λυπημένη				
19. Νιώθετε ότι οι άνθρωποι δεν σας συμπαθούν				

α. λιγότερο από 1 μέρα την εβδομάδα = «σπάνια, καθόλου»
β. 1-2 μέρες = «μερικές φορές»
γ. 3-4 μέρες = «αρκετές φορές»
δ. 5-7 μέρες = «τον περισσότερο καιρό»

Τις τελευταίες 7 μέρες :

20. Δεν μπορείτε να κάνετε τις δουλειές σας (εργασία, σπίτι, σχολείο)				

ΕΡΩΤΗΜΑΤΟΛΟΓΙΟ ΤΩΝ ΠΕΝΤΕ ΠΑΡΑΓΟΝΤΩΝ ΤΗΣ ΠΡΟΣΩΠΙΚΟΤΗΤΑΣ (Big Five Inventory-BFI)

		**Διαφωνώ απόλυτα**	**Διαφωνώ λίγο**	**Ουδέτερο**	**Συμφωνώ λίγο**	**Συμφωνώ Απόλυτα**
1	Είναι ομιλητικός	1	2	3	4	5
2	Τείνει να βρίσκει σφάλματα σε άλλους	1	2	3	4	5
3	Κάνει μια δουλειά πολύ προσεκτικά	1	2	3	4	5
4	Είναι μελαγχολικός, άκεφος	1	2	3	4	5
5	Είναι πρωτότυπος, βρίσκει όλο καινούριες ιδέες	1	2	3	4	5
6	Είναι επιφυλακτικός	1	2	3	4	5
7	Είναι βοηθητικός, και ανιδιοτελής με τους άλλους	1	2	3	4	5

8	Μπορεί να είναι κάπως απρόσεκτος	1	2	3	4	5

Στο ερωτηματολόγιο αυτό θα βρείτε κάποια χαρακτηριστικά της προσωπικότητας τα οποία μπορεί να ταιριάζουν ή να μην ταιριάζουν σε σας. Παρακαλούμε κυκλώστε τον αριθμό δίπλα από κάθε πρόταση που ακολουθεί, ο οποίος να αντιστοιχεί στο βαθμό που συμφωνείτε ή διαφωνείτε με τη συγκεκριμένη πρόταση.

1: Διαφωνώ απόλυτα, 2. Διαφωνώ λίγο, 3. Ουδέτερο, 4. Συμφωνώ λίγο, 5. Συμφωνώ Απόλυτα

Βλέπω τον εαυτό μου σαν κάποιον που …

9	Είναι ήρεμος, αντιμετωπίζει το άγχος καλά	1	2	3	4	5
10	Είναι περίεργος για πολλά διαφορετικά πράγματα	1	2	3	4	5
11	Είναι γεμάτος ενεργητικότητα	1	2	3	4	5
12	Ξεκινά φιλονικίες με άλλους	1	2	3	4	5
13	Είναι ένας αξιόπιστος εργαζόμενος	1	2	3	4	5
14	Μπορεί να βρίσκεται σε ένταση	1	2	3	4	5
15	Είναι ευφυής, ένας βαθύς στοχαστής	1	2	3	4	5
16	Προκαλεί πολύ ενθουσιασμό	1	2	3	4	5
17	Από τη φύση του συγχωρεί	1	2	3	4	5
18	Έχει την τάση να είναι ανοργάνωτος	1	2	3	4	5
19	Ανησυχεί πολύ	1	2	3	4	5
20	Έχει ζωηρή φαντασία	1	2	3	4	5

21	Έχει την τάση να είναι ήσυχος	1	2	3	4	5
22	Είναι γενικώς άτομο εμπιστοσύνης	1	2	3	4	5

23	Έχει την τάση να είναι νωχελικός	1	2	3	4	5
24	Έχει συναισθηματική σταθερότητα, δεν εκνευρίζεται εύκολα	1	2	3	4	5
25	Είναι εφευρετικός	1	2	3	4	5
26	Εμπνέει μια σιγουριά	1	2	3	4	5
27	Μπορεί να είναι ψυχρός και ακατάδεκτος	1	2	3	4	5
28	Επιμένει μέχρι να τελειώσει το έργο που κάνει	1	2	3	4	5
29	Μπορεί να είναι κακόκεφος	1	2	3	4	5
30	Εκτιμά καλλιτεχνικές, αισθητικές εμπειρίες	1	2	3	4	5
31	Μερικές φορές είναι ντροπαλός, έχει αναστολές	1	2	3	4	5

32	Είναι διακριτικός και ευγενικός σχεδόν με όλους	1	2	3	4	5

33	Κάνει τα πράγματα με αποτελεσματικότητα	1	2	3	4	5
34	Παραμένει ήρεμος σε καταστάσεις έντασης	1	2	3	4	5
35	Προτιμά τη δουλειά που είναι ρουτίνα	1	2	3	4	5
36	Του αρέσει να βγαίνει έξω, είναι κοινωνικός	1	2	3	4	5
37	Μερικές φορές είναι αγενής προς τους άλλους	1	2	3	4	5
38	Κάνει σχέδια και τα ακολουθεί	1	2	3	4	5
39	Νευριάζει εύκολα	1	2	3	4	5
40	Του αρέσει να συλλογίζεται, να παίζει με ιδέες	1	2	3	4	5
41	Έχει λίγα καλλιτεχνικά ενδιαφέροντα	1	2	3	4	5
42	Του αρέσει να συνεργάζεται με τους άλλους	1	2	3	4	5

43	Αποσπάται εύκολα η προσοχή του	1	2	3	4	5
44	Είναι γνώστης της τέχνης, της μουσικής, της λογοτεχνίας	1	2	3	4	5

Multiple Sclerosis Quality of Life
Ποιότητα ζωής σε ασθενείς με Πολλαπλή Σκλήρυνση
(MSQOL)-54 Instrument

Barbara G. Vickrey, MD, MPH

Copyright© 1995, University of California, Los Angeles

Ημερομηνία……………………………………………………………….

<u>ΟΔΗΓΙΕΣ</u>

Στην έρευνα αυτή οι ερωτήσεις αφορούν την υγεία σας και τις καθημερινές σας δραστηριότητες. <u>Απαντήστε σε όλες</u> τις ερωτήσεις βάζοντας σε κύκλο τον αντίστοιχο αριθμό (1, 2, 3, …)

1. Γενικά, θα χαρακτηρίζατε την υγεία σας ως: (κυκλώστε ένα αριθμό)

Άριστη .... 1 Πολύ

καλή 2

Καλή 3

Μέτρια 4

Άσχημη 5

2. <u>Συγκριτικά με ένα χρόνο πριν</u>, πώς θα αξιολογούσατε την υγεία σας <u>τώρα</u>; (κυκλώστε ένα αριθμό)

Πολύ καλύτερη τώρα από ότι πριν ένα χρόνο 1

Κάπως καλύτερη τώρα από ότι πριν ένα χρόνο 2

Σχεδόν το ίδιο 3

Κάπως χειρότερα τώρα από ότι ένα χρόνο πριν 4

Πολύ χειρότερα τώρα από ότι ένα χρόνο πριν 5

3-12. Οι ερωτήσεις που ακολουθούν αφορούν δραστηριότητες που πιθανόν να περιλαμβάνει μια τυπική ημέρα σας. Σας περιορίζει **η υγεία σας** σε αυτές τις δραστηριότητες; Αν ναι σε ποιο βαθμό; (κυκλώστε 1,2 ή 3 σε κάθε σειρά)

	Ναι<br>Με περιορίζει πολύ	Ναι<br>Με περιορίζει λίγο	Όχι<br>Δε με περιορίζει Καθόλου
3. Έντονες δραστηριότητες, όπως το τρέξιμο, η ανύψωση βαρέων αντικειμένων, η ενασχόληση με αθλήματα που απαιτούν έντονη προσπάθεια	1	2	3
4.Μέτριας έντασης δραστηριότητες, όπως η μετακίνηση ενός τραπεζιού το να σπρώχνετε μία ηλεκτρική σκούπα, το Bowling, το Golf	1	2	3
5. Το ανασήκωμα ή η μεταφορά μαναβικής	1	2	3
6. Η άνοδος αρκετών ορόφων με τις σκάλες	1	2	3
7. Η άνοδος ενός ορόφου με τις σκάλες	1	2	3
8. Να σκύβετε, να γονατίζετε ή να καμπουριάζετε	1	2	3
9. Το περπάτημα απόστασης μεγαλύτερης από 1600m.	1	2	3
10.Το περπάτημα αρκετών οικοδομικών τετραγώνων	1	2	3

11.Το περπάτημα ενός οικοδομικού τετραγώνου	1	2	3
12.Το να πλένεστε και να ντύνεστε μόνος σας	1	2	3

13-16. Κατά τη διάρκεια των **προηγούμενων τεσσάρων εβδομάδων**, είχατε κάποιο από τα παρακάτω προβλήματα στην εργασία σας ή σε κάποιες άλλες δραστηριότητες της συνηθισμένης καθημερινής σας ζωής, **που οφείλονται σε προβλήματα στη σωματική σας υγεία**; (κυκλώστε ένα αριθμό σε κάθε σειρά)

	ΝΑΙ	ΟΧΙ
13. Μείωση του χρόνου που μπορείτε να αφιερώσετε στην εργασία ή σε άλλες δραστηριότητες	1	2
14. Πετύχατε λιγότερα από όσα θα θέλατε	1	2
15. Περιοριστήκατε σε κάποιο είδος εργασίας ή σε άλλες δραστηριότητες.	1	2
16. Είχατε δυσκολία στην εκτέλεση κάποιας εργασίας ή κάποιας άλλης δραστηριότητας. (για παράδειγμα χρειάστηκε μεγαλύτερη προσπάθεια)	1	2

17-19. Κατά τη διάρκεια των προηγούμενων τεσσάρων εβδομάδων, είχατε κάποιο από τα παρακάτω προβλήματα στην εργασία σας ή σε κάποιες άλλες δραστηριότητες της συνηθισμένης καθημερινής σας ζωής, που οφείλονται σε συναισθηματικές διαταραχές; (για παράδειγμα να νιώθατε κατάθλιψη ή άγχος;) (κυκλώστε ένα αριθμό σε κάθε σειρά)

	ΝΑΙ	ΟΧΙ
17. Μείωση του χρόνου που μπορείτε να αφιερώσετε στην εργασία ή σε άλλες δραστηριότητες	1	2

18. <u>Πετύχατε λιγότερα από όσα θα θέλατε</u>	1	2
19. Η εργασία ή άλλες δραστηριότητες δεν έγιναν από εσάς τόσο <u>προσεκτικά</u> όσο συνήθως	1	2

20. Κατά τη διάρκεια των **<u>προηγούμενων τεσσάρων εβδομάδων</u>**, σε ποιο βαθμό επηρέασε η σωματική σας υγεία ή οι συναισθηματικές σας διαταραχές τις φυσιολογικές κοινωνικές σας δραστηριότητες με την οικογένεια, τους φίλους, τους γείτονες ή άλλες ομάδες; (κυκλώστε ένα αριθμό)

Καθόλου 1

Ελαφρώς 2

Μετρίως 3

Αρκετά 4

Υπερβολικά Πολύ 5

ΠΟΝΟΣ

21. Πόσο **<u>σωματικό πόνο</u>** νιώσατε κατά τη διάρκεια των **<u>προηγούμενων τεσσάρων εβδομάδων;</u>** (κυκλώστε ένα αριθμό)

Καθόλου 1

Πολύ ήπιο 2

Ήπιο 3

Μέτριο 4

Έντονο ....5 Πολύ

έντονο 6

22. Κατά τη διάρκεια των προηγούμενων **τεσσάρων εβδομάδων**, πόσο επηρέασε **ο πόνος** την κανονική εργασία σας (τόσο την εργασία εκτός οικίας όσο και την οικιακή εργασία);
(κυκλώστε ένα αριθμό)

Καθόλου 1

Πολύ λίγο 2

Μετρίως 3

Αρκετά 4

Υπερβολικά πολύ 5

23-32. Οι ερωτήσεις που ακολουθούν αφορούν το πώς αισθάνεστε καθώς και το πώς ήταν η κατάστασή σας, **κατά τη διάρκεια των προηγούμενων τεσσάρων εβδομάδων.** Σε κάθε ερώτηση παρακαλώ να δώσετε την απάντηση που περιγράφει καλύτερα το πώς νιώθατε.

Πόσο χρόνο από αυτές τις **τέσσερις προηγούμενες εβδομάδες…….**
(κυκλώστε ένα αριθμό σε κάθε σειρά)

	Συνεχώς	Τον περισσότερο χρόνο	Ένα σημαντικό Χρονικό διάστημα	Κάποιο χρονικό διάστημα	Μικρό χρονικό διάστημα	Καθόλου
23. Αισθανόσασταν δραστήριος;	1	2	3	4	5	6
24.Ήσασταν νευρικός;	1	2	3	4	5	6

25. Νιώθατε τόσο στεναχωρημένος που τίποτε δεν μπορούσε να σας κάνει να χαρείτε;	1	2	3	4	5	6
26.Νιώθατε ήρεμος και γαλήνιος;	1	2	3	4	5	6
27.Είχατε πολλή ενέργεια;	1	2	3	4	5	6
28.Νιώθατε αποκαρδιωμένος και μελαγχολικός	1	2	3	4	5	6
29. Νιώθατε εξαντλημένος	1	2	3	4	5	6
30.Ήσασταν χαρούμενος;	1	2	3	4	5	6
31. Νιώθατε κουρασμένος;	1	2	3	4	5	6
32. Νιώθατε ξεκούραστος κατά την πρωινή έγερση;	1	2	3	4	5	6

33. Κατά τη διάρκεια των **προηγούμενων τεσσάρων εβδομάδων**, για πόσο μεγάλο χρονικό διάστημα επηρέασε **η σωματική σας υγεία ή οι συναισθηματικές σας διαταραχές** την κοινωνική σας δραστηριότητα (όπως τις επισκέψεις σε φίλους, συγγενείς κτλ); (Κυκλώστε ένα αριθμό)

Συνεχώς 1

Τον περισσότερο χρόνο .... 2 Κάποιο

χρονικό διάστημα .... 3 Μικρό χρονικό

διάστημα 4

Καθόλου 5

ΓΕΝΙΚΑ ΓΙΑ ΤΗΝ ΥΓΕΙΑ

34-37. Πόσο ΣΩΣΤΗ ή ΛΑΘΟΣ είναι για σας κάθε μια από τις παρακάτω δηλώσεις (κυκλώστε ένα αριθμό σε κάθε σειρά)

	Σίγουρα σωστή	Το περισσότερο σωστή	Όχι Σίγουρος	Το περισσότερο λανθασμένη	Σίγουρα Λάθος
34.Φαίνεται πως αρρωσταίνω λίγο ευκολότερα από άλλους	1	2	3	4	5
35.Είμαι τόσο υγιής όσο κάθε άνθρωπος που γνωρίζω	1	2	3	4	5
36.Περιμένω πως η υγεία μου θα χειροτερεύσει.	1	2	3	4	5
37.Η υγεία μου είναι άριστη	1	2	3	4	5

ΑΝΗΣΥΧΙΑ ΓΙΑ ΤΗΝ ΥΓΕΙΑ

Πόσο χρόνο κατά **τη διάρκεια των προηγούμενων τεσσάρων εβδομάδων….**

(κυκλώστε ένα αριθμό σε κάθε σειρά)

	Συνεχώς	Τον περισσότερο χρόνο	Ένα σημαντικό Χρονικό διάστημα	Κάποιο χρονικό διάστημα	Μικρό χρονικό διάστημα	Καθόλου
38.Αποθαρυνθήκατε λόγω των προβλημάτων υγείας σας;	1	2	3	4	5	6
39.Ήσασταν αγανακτισμένος λόγω της υγείας σας;	1	2	3	4	5	6
40.Σας απασχολούσε το θέμα της υγείας σας	1	2	3	4	5	6
41.Νιώθατε πολύ πεσμένος λόγω των προβλημάτων της υγείας σας.	1	2	3	4	5	6

ΓΝΩΣΤΙΚΗ ΛΕΙΤΟΥΡΓΙΑ

Πόσο χρόνο κατά **τη διάρκεια των προηγούμενων τεσσάρων εβδομάδων….**

(κυκλώστε ένα αριθμό σε κάθε σειρά)

	Συνεχώς	Τον περισσότερο χρόνο	Ένα σημαντικό Χρονικό διάστημα	Κάποιο χρονικό διάστημα	Μικρό χρονικό διάστημα	Καθόλου
42.Είχατε δυσκολία συγκέντρωσης και σκέψης;	1	2	3	4	5	6
43.Είχατε πρόβλημα στη διατήρηση της προσοχής σας σε κάποια δραστηριότητα για αρκετή ώρα;	1	2	3	4	5	6
44.Είχατε πρόβλημα με τη μνήμη σας;	1	2	3	4	5	6
45.Πρόσεξαν οι συγγενείς ή οι φίλοι σας ότι είχατε πρόβλημα με τη μνήμη σας ή πρόβλημα στη συγκέντρωση;	1	2	3	4	5	6

ΣΕΞΟΥΑΛΙΚΗ ΛΕΙΤΟΥΡΓΊΑ

46-50. Οι ερωτήσεις που ακολουθούν αφορούν τη σεξουαλική σας λειτουργία και το πόσο ευχαριστημένος είστε από αυτή. Παρακαλώ απαντήστε όσο το δυνατόν σαφέστερα, **για το διάστημα των τεσσάρων τελευταίων εβδομάδων μόνο**.

Πόσο μεγάλο πρόβλημα ήταν για εσάς κάθε ένα από τα παρακάτω, **στο χρονικό διάστημα των τεσσάρων τελευταίων εβδομάδων;**

(κυκλώστε ένα αριθμό σε κάθε σειρά)

**ΓΥΝΑΙΚΕΣ**	Κανένα πρόβλημα	Μικρό πρόβλημα	Μέτριο πρόβλημα	Μεγάλο πρόβλημα
46. Έλλειψη σεξουαλικού ενδιαφέροντος	1	2	3	4
47.Ανεπαρκής λίπανση	1	2	3	4
48.Δυσκολία στον οργασμό	1	2	3	4
49.Ικανότητα να ικανοποιήσετε τον ερωτικό σύντροφο	1	2	3	4

50. Γενικά πόσο ικανοποιημένος ήσασταν από τη σεξουαλική σας δραστηριότητα **κατά τη διάρκεια των τεσσάρων τελευταίων εβδομάδων**; (Κυκλώστε ένα αριθμό)

Πολύ ικανοποιημένος /η 1

Κάπως ικανοποιημένος /η 2

Ούτε ικανοποιημένος /η Ούτε δυσαρεστημένος /η 3

Κάπως δυσαρεστημένος /η 4

Πολύ δυσαρεστημένος /η 5

51. Κατά τη διάρκεια **των τεσσάρων τελευταίων εβδομάδων**, σε ποιο βαθμό επηρέασαν τα προβλήματα ούρησης και αφόδευσης τις φυσιολογικές κοινωνικές σας δραστηριότητες με την οικογένεια, τους φίλους, τους γείτονες, ή άλλες κοινωνικές ομάδες; (Κυκλώστε ένα αριθμό)

Καθόλου .... 1 Ελαφρώς

.... 2

Μέτρια 3

Αρκετά 4

Πάρα πολύ 5

52. Κατά τη διάρκεια των τελευταίων τεσσάρων εβδομάδων πόσο επηρέασε ο πόνος την ευχαρίστηση που αντλείτε από τη ζωή;

(Κυκλώστε ένα αριθμό)

Καθόλου 1
Ελαφρώς 2
Μέτρια 3
Αρκετά 4
Πάρα πολύ 5

Γενικά πως θα αξιολογούσατε την ποιότητα της ζωής σας:

Επιλέξτε ένα αριθμό στην κλίμακα που ακολουθεί:

0	1	2	3	4	5	6	7	8	9	10

Χειρότερη δυνατή ποιότητα ζωής. *Τόσο άσχημα ή και χειρότερα από το να έχω πεθάνει*

Καλύτερη δυνατή ποιότητα ζωής

53.

54. Ποιο από τα παρακάτω περιγράφει καλύτερα τα συναισθήματά σας για τη ζωή σας ως σύνολο; (Κυκλώστε ένα αριθμό)

Φρικτή 1

Δυστυχισμένη .... 2 Κυρίως

μη ικανοποιημένος/ η... 3

Ανάμεικτα, σχεδόν το ίδιο,
Ικανοποιημένος /η και δυσαρεστημένος/ η 4
Κατά κύριο λόγο ικανοποιημένος /η 5

Ευχαριστημένος /η 6

Ευτυχισμένος /η 7

Motivation for Parenthood Questionnaire (PM2).

Υποδείξτε την απάντηση που πιστεύετε ότι είναι η καλύτερη τοποθετώντας ένα (1) μπροστά της. Κατατάξτε τις υπόλοιπες απαντήσεις (2, 3, 4) για να εμφανίσετε τη σειρά προτίμησής σας.

1. Οι γονείς προσδοκούν τα παιδιά τους. . .. . . ☐ *Να εκπληρώσουν το σκοπό της ζωής*
   - ☐ *Να δυναμώσουν την οικογένεια*
   - ☐ *Να είναι υγιή και χαρούμενα*
   - ☐ *Να ακολουθήσουν τα βήματά τους*

2. *Οι άντρες θέλουν παιδιά γιατί. . .*
   - ☐ *Θα ήθελαν να αποδείξουν τη σεξουαλική τους επάρκεια*
   - ☐ *Είναι φυσικό ένστικτο*
   - ☐ *Τα χρειάζονται για να ενισχύσουν την κοινωνική τους θέση*
   - ☐ *Τους αρέσουν τα παιδιά*

3. *Ο έλεγχος των γεννήσεων. . .*
   - ☐ *Είναι απαραίτητο μέτρο*
   - ☐ *Είναι εγγενώς λάθος*
   - ☐ *Απαιτεί τη συμφωνία του συζύγου και της συζύγου*
   - ☐ *Δεν είναι φυσικό*

4. *Η μητέρα προσδοκά την κόρη της. . .*
   - ☐ *Να της προσφέρει συντροφιά και στοργή*
   - ☐ *Να πάρει τη θέση στον κόσμο για τον οποίο είναι προορισμένη*
   - ☐ *Να είναι όπως ο εαυτός της*
   - ☐ *Να είναι χαρούμενη και καλά*

5. *Οι άντρες θέλουν παιδιά γιατί. . .*
   - ☐ *Τα παιδιά δυναμώνουν το γάμο*
   - ☐ *Τους αρέσει να φροντίζουν και να παρέχουν στα παιδιά*
   - ☐ *Είναι συνάρτηση του ώριμου ενήλικα*
   - ☐ *Θέλουν να διαιωνιστούν*

6. *Ο πατέρας προσδοκά τον γιο του. . .   Να είναι χαρούμενος και καλά*
   - ☐ ☐ *Να πάρει τη θέση στον κόσμο για τον οποίο είναι προορισμένος*
   - ☐
   - ☐

Να του δώσει συντροφιά και στοργή

Να είναι όπως ο εαυτός του

7. *Οι γυναίκες θέλουν παιδιά γιατί. . .*
   - *Τους αρέσουν τα παιδιά*
   - *Τα χρειάζονται για να ενισχύσουν την κοινωνική τους θέση*
   - *Θα ήθελαν να αποδείξουν τη σεξουαλική τους επάρκεια*
   - *Είναι φυσικό ένστικτο*

8. *Η Προγραμματισμένη γονεϊκότητα*
   - *Είναι αδύνατη*
   - *Πρέπει να λαμβάνεται υπόψη από όλους*
   - *Είναι το πιο επιθυμητό*
   - *Οδηγεί σε προβλήματα*

9. *Γενικά, οι άνθρωποι θέλουν παιδιά επειδή. . .*
   - *Προορίζονται να αναπαραχθούν*
   - *Επιθυμούν να βοηθήσουν κάποιον να μεγαλώσει και να αναπτυχθεί*
   - *Δημιουργούν κάποιον κατ' εικόνα τους*
   - *Παρέχουν συντροφικότητα*

10. *Ο πατέρας προσδοκά την κόρη του.. . .*
    - *Να πιστέψει σε αυτόν*
    - *Να είναι χαρούμενη και καλά*
    - *Να πάρει τη θέση της στον κόσμο*
    - *Να του δώσει συντροφιά και στοργή*

11. *Οι γυναίκες θέλουν παιδιά γιατί. . .*
    - *Τα παιδιά δυναμώνουν το γάμο*
    - *Είναι συνάρτηση του ώριμου ενήλικα*
    - *Τους αρέσει να φροντίζουν και να παρέχουν στα παιδιά*
    - *Θέλουν να διαιωνιστούν*

12. *Οι μεγάλες οικογένεις . . .*
    - *Είναι ευτυχισμένες*
    -
    -
    -

Προκαλούν μεγάλα προβλήματα

Είναι απαρχαιωμένες

Είναι πιο δεμένες από τις μικρές οικογένειες

13. *Οι γυναίκες θέλουν παιδιά γιατί.. .*

☐ *Προορίζονται να αναπαραχθούν*

☐ *Επιθυμούν να βοηθήσουν κάποιον να μεγαλώσει και να αναπτυχθεί*

☐ *Παρέχουν συντροφικότητα*

☐ *Δημιουργούν κάποιον κατ' εικόνα τους*

14. *Γενικά, οι άνθρωποι θέλουν παιδιά επειδή. . .*

☐ *Τους αρέσει να φροντίζουν και να παρέχουν στα παιδιά*

☐ *Θέλουν να διαιωνιστούν*

☐ *Τα παιδιά δυναμώνουν το γάμο*

☐ *Είναι συνάρτηση του ώριμου ενήλικα*

15. *Η μητέρα προσδοκά τον γιο της. . .*

☐ *Να πάρει τη θέση του στον κόσμο*

☐ *Να της δώσει συντροφιά και στοργή*

☐ *Να είναι χαρούμενος και καλά*

☐*Να πιστέψει σε αυτήν*

16. *Οι άντρες θέλουν παιδιά γιατί. . .*

☐ *Παρέχουν συντροφικότητα*

☐ *Δημιουργούν κάποιον κατ' εικόνα τους*

☐ *Προορίζονται να αναπαραχθούν*

☐ *Επιθυμούν να βοηθήσουν κάποιον να μεγαλώσει και να αναπτυχθεί*

17. *Παιδιά που δεν έχουν προγραμματιστεί. . .*

☐ *Είναι μια ευπρόσδεκτη ευλογία*

☐ *Δύσκολα γίνονται αποδεκτά*

☐ *Αγαπιούνται και λατρεύονται*

☐ *Είναι μερικές φορές ανεπιθύμητα*

18. *Γενικά, οι άνθρωποι θέλουν παιδιά επειδή. . .*

- *Είναι φυσικό ένστικτο*
- *Τους αρέσουν τα παιδιά*
- *Τα χρειάζονται για να ενισχύσουν την κοινωνική τους θέση*
- *Θα ήθελαν να αποδείξουν τη σεξουαλική τους επάρκεια*

The Positivity Scale

Οι παρακάτω δηλώσεις περιγράφουν πώς ένα άτομο μπορεί να ενεργήσει σε ορισμένες καταστάσεις.

Παρακαλούμε κυκλώστε τον αριθμό δίπλα από κάθε πρόταση που ακολουθεί, ο οποίος να αντιστοιχεί στο βαθμό που συμφωνείτε ή διαφωνείτε με τη συγκεκριμένη πρόταση.

	**Συμφωνώ Απόλυτα**	**Συμφωνώ**	**Ούτε Συμφωνώ Ούτε Διαφωνώ**	**Διαφωνώ**	**Διαφωνώ Απόλυτα**
1. Έχω μεγάλη πίστη στο μέλλον					
2. Είμαι ικανοποιημένος/η με τη ζωή μου					
3. Οι άλλοι είναι γενικά εδώ για μένα όταν τους χρειάζομαι					
4. Προσβλέπω στο μέλλον με ελπίδα και ενθουσιασμό					
5. Σε γενικές γραμμές, είμαι ικανοποιημένος/η με τον εαυτό μου					
6. Μερικές φορές, το μέλλον μου φαίνεται ασαφές (αντίστροφη βαθμολογία)					
7. Νιώθω ότι έχω πολλά πράγματα για τα οποία να είμαι περήφανος/η					
8. Γενικά νιώθω σίγουρος/η για τον εαυτό μου					

Supplementary Table 1. Association between parity and additional psychosocial, personality, quality-of-life, and motivational variables among women with multiple sclerosis in Athens, Greece (August 2023–December 2024; n = 103) (Part II).

	**Number of children**

Parameter	0	1	2 Mean value (standard deviation)	p
Dyadic Consensus Scale	20.22 (5.54)	21.00 (5.46)	21.52 (3.87)	0.636
Dyadic Satisfaction Scale	28.35 (1.90)	28.39 (2.39)	28.48 (1.87)	0.976
Dyadic Cohesion Scale	15.61 (2.20)	15.02 (2.16)	15.28 (2.26)	0.279
Affectional Expression Scale	3.35 (1.25)	3.52 (1.36)	3.92 (1.38)	0.514
BFI- Extraversion	22.48 (3.11)	23.17 (3.68)	22.32 (3.15)	0.572
BFI- Agreeableness	30.96 (3.03)	32.32 (2.95)	30.80 (3.57)	0.075
BFI- Conscientiousness	29.70 (4.72)	30.43 (3.52)	30.72 (5.25)	0.659
BFI- Neuroticism	25.77 (4.69)	24.56 (4.01)	24.28 (2.44)	0.254
BFI- Openness	32.83 (4.68)	31.45 (4.28)	32.00 (3.96)	0.394
MSQOL-54- Physical Function	43.22 (19.64)	42.44 (12.64)	44.20 (18.18)	0.912
MSQOL-54- Role Limitations Due to Physical Problems	11.71 (25.38)	13.33 (20.36)	15.00 (22.82)	0.862
MSQOL-54- Role Limitations Due to Emotional Problems	23.95 (27.08)	29.62 (25.84)	24.00 (28.08)	0.573
MSQOL-54-Pain	46.04 (18.98)	43.07 (10.46)	44.66 (9.81)	0.638
MSQOL-54-Social Function	50.87 (14.01)	54.66 (12.12)	60.48 (12.12)	**0.021**
MSQOL-54- Energy	38.50 (12.84)	42.04 (12.04)	41.76 (12.60)	0.432
MSQOL-54- Health Perceptions	26.71 (12.08)	28.66 (8.88)	38.00 (18.92)	**0.003**
MSQOL-54- Social Function	57.77 (18.03)	62.59 (12.24)	65.94 (13.27)	0.122

MSQOL-54- Cognitive Function	71.25 (21.28)	76.22 (17.32)	78.40 (10.07)	0.267
MSQOL-54-Health Distress	44.06 (23.08)	55.11 (19.11)	60.80 (11.51)	**0.004**
MSQOL-54-Sexual Function	49.48 (29.26)	42.60 (23.52)	48.99 (19.90)	0.403
PM2-Altruistic	39.16 (3.58)	36.66 (5.52)	37.16 (3.48)	0.393
PM2-Fatalistic	44.08 (3.50)	48.77 (2.48)	46.50 (3.20)	**0.009**
PM2-Narcissistic	45.83 (1.94)	42.88 (4.16)	45.66 (2.94)	0.088
PM2-Instrumental	51.81 (3.57)	52.11 (3.51)	51.00 (1.54)	0.803

BFI: Big Five Inventory; MSQOL: Multiples Sclerosis Quality of Life

Continuous variables were compared using ANOVA. **Data are presented as mean (standard deviation); statistical significance was set at $p < 0.05$**

Supplementary Table 2. Comparison between women with no pregnancy and those with ≥1 pregnancy among women with multiple sclerosis in Athens, Greece (August 2023–December 2024; n = 103).

**Parameter**	**No pregnancy (n = 32)**	**≥1 pregnancy (n = 71)**	**p-value**
Age (years), mean (SD)	35.56 (8.67)	40.20 (6.01)	0.017
Currently on DMT (%)	84.4%	56.3%	0.028
Disease course (RRMS) (%)	87.5%	88.7%	0.91
Disability (EDSS), median (IQR)	1.5 (1.0–2.0)	1.5 (1.0–2.5)	0.76
Years since MS diagnosis (n, %)	—	—	0.044
Beta-interferon use (%)	53.3% (No) vs 21.9% (Yes)	36.7% (No) vs 47.9% (Yes)	0.006
Fingolimod use (%)	100% (4/4) on fingolimod were in this group	0%	0.011
MSQOL-54: Mental health score, mean (SD)	48.56 (16.76)	54.97 (12.05)*	0.100
MSQOL-54: Health perceptions	26.71 (12.08)	31.31 (13.16)*	0.003
Multivariable analysis	—	—	No independent predictors

Between-group comparisons were conducted using independent-samples t-tests for continuous variables and chi-square or Fisher’s exact tests for categorical variables;

disability (EDSS) was compared using the Mann–Whitney U test because it is reported as median (IQR). **Data are presented as mean (standard deviation), median (interquartile range), or n (%); statistical significance was set at p < 0.05.**

Supplementary Table 3. Comparison between parous women with one child and those with ≥2 children among women with multiple sclerosis in Athens, Greece (August 2023–December 2024; n = 71).

**Parameter**	**One child (n = 41)**	**≥2 children (n = 30)**	**p-value**
Age (years), mean (SD)	37.72 (5.02)	43.48 (5.10)	**0.001**
MSQOL-54 Mental Health Score	53.97 (13.63)	56.23 (10.47)	0.100
MSQOL-54 Health perceptions	28.66 (8.88)	38.00 (18.92)	**0.003**
MSQOL-54 Social function	54.66 (12.12)	60.48 (12.12)	**0.021**
MSQOL-54 Health distress	55.11 (19.11)	60.80 (11.51)	**0.004**
Beta-interferon use (%)	47.9%	30.1%	0.006
Fingolimod use (%)	0%	0%	—
Marital status (married, %)	53.1%	30.9%	0.001
MS disease progression category	RRMS: 48.9%	RRMS: 25%	0.005
Multivariable analysis	—	—	No independent predictors

Between-group comparisons were conducted using independent-samples t-tests for continuous variables and chi-square or Fisher's exact tests for categorical variables. **Data are presented as mean (standard deviation) or n (%); statistical significance was set at p < 0.05.**

Supplementary Table 4. Association between attempts to conceive and additional psychosocial, personality, quality-of-life, and motivational variables among childless women with multiple sclerosis in Athens, Greece (August 2023–December 2024; n = 32) (Part II).

**Parameter**	**Attempted conception**		**p**
	**No**	**Yes**	
	**Mean value (standard deviation)**		
Dyadic Consensus Scale	21.00 (5.53)	19.00 (5.55)	0.338
Dyadic Satisfaction Scale	28.26 (2.15)	28.50 (1.50)	0.742
Dyadic Cohesion Scale	3.73 (1.14)	2.75 (1.21)	0.954
Affectional Expression Scale	15.63 (2.45)	15.58 (1.83)	**0.030**
BFI- Extraversion	22.63 (3.14)	22.25 (3.19)	0.746
BFI- Agreeableness	30.84 (3.46)	31.16 (2.32)	0.778
BFI- Conscientiousness	29.63 (5.15)	29.83 (4.15)	0.910
BFI- Neuroticism	26.05 (4.93)	25.33 (4.45)	0.685
BFI- Openness	32.47 (4.71)	33.41 (4.77)	0.594

MSQOL-54- Physical Function	45.55 (18.77)	40.00 (21.11)	0.447
MSQOL-54- Role Limitations Due to Physical Problems	6.57 (16.33)	19.23 (34.08)	0.232
MSQOL-54- Role Limitations Due to Emotional Problems	21.05 (22.79)	28.20 (32.90)	0.472
MSQOL-54-Pain	48,07 (17.54)	43.07 (21.28)	0.474
MSQOL-54-Social Function	52.00 (11.69)	49.23 (17.23)	0.619
MSQOL-54- Energy	41.47 (12.52)	34.15 (12.50)	0.115
MSQOL-54- Health Perceptions	25.78 (10.96)	28.07 (13.92)	0.607
MSQOL-54- Social Function	58.77 (19.31)	56.06 (18.29)	0.699
MSQOL-54- Cognitive Function	70.78 (18.94)	71.92 (25.12)	0.885
MSQOL-54-Health Distress	45.00 (20.94)	42.69 (26.74)	0.786
MSQOL-54-Sexual Function	49.56 (31.61)	49.35 (26.68)	0.984
PM2-Altruistic	38.50 (3.33)	40.50 (4.20)	0.388
PM2-Fatalistic	44.87 (3.64)	42.50 (3.00)	0.289
PM2-Narcissistic	45.87 (2.03)	45.75 (2.06)	0.924
PM2-Instrumental	51.85 (3.48)	51.75 (4.27)	0.965

BFI: Big Five Inventory; MSQOL: Multiples Sclerosis Quality of Life

Continuous variables were compared using independent-samples t-test. **Data are presented as mean (standard deviation); statistical significance was set at $p < 0.05$**

Supplementary Table 5. Correlations between Motivation for Parenthood (PM2) subscale scores and clinical, psychological, and quality-of-life parameters among women with multiple sclerosis in Athens, Greece (August 2023–December 2024; n = 103).

Variable	Altruistic r (p)	Fatalistic r (p)	Narcissistic r (p)	Instrumental r (p)
Age (years)	−0.321 (0.103)	0.311 (0.114)	−0.061 (0.762)	−0.154 (0.454)
OSSS-3	−0.179 (0.392)	0.229 (0.271)	−0.053 (0.800)	0.060 (0.781)
Dyadic Adjustment Scale (total)	−0.461 (0.016)	0.504 (0.007)	−0.058 (0.775)	0.113 (0.583)
Dyadic Consensus	−0.435 (0.023)	0.486 (0.010)	−0.149 (0.459)	0.171 (0.405)
Dyadic Satisfaction	0.044 (0.829)	0.043 (0.833)	0.093 (0.643)	−0.208 (0.309)
Dyadic Cohesion	−0.440 (0.022)	0.560 (0.002)	−0.352 (0.072)	0.262 (0.196)
Affectional Expression	0.068 (0.738)	−0.215 (0.281)	0.302 (0.126)	−0.071 (0.730)
CES-D score	0.199 (0.320)	0.006 (0.978)	−0.123 (0.540)	−0.185 (0.365)
BFI – Extraversion	0.380 (0.050)	−0.238 (0.232)	−0.106 (0.598)	−0.186 (0.364)
BFI – Agreeableness	0.264 (0.182)	−0.156 (0.437)	0.020 (0.921)	−0.209 (0.305)
BFI – Conscientiousness	0.450 (0.019)	−0.272 (0.169)	−0.235 (0.239)	−0.189 (0.354)

BFI – Neuroticism	−0.154 (0.444)	−0.033 (0.868)	0.245 (0.219)	0.116 (0.571)
BFI – Openness	0.284 (0.151)	−0.201 (0.315)	0.087 (0.667)	−0.188 (0.359)
BFI – Overall score	0.463 (0.015)	−0.341 (0.082)	0.000 (0.998)	−0.240 (0.237)
MSQOL-54 Physical function	−0.324 (0.099)	0.412 (0.033)	0.047 (0.815)	−0.121 (0.557)
MSQOL-54 Pain	−0.230 (0.248)	0.388 (0.046)	0.131 (0.516)	−0.294 (0.145)
MSQOL-54 Energy	−0.199 (0.319)	0.433 (0.024)	−0.109 (0.589)	−0.135 (0.512)
MSQOL-54 Social function	−0.299 (0.146)	0.465 (0.019)	−0.051 (0.809)	−0.021 (0.924)
MSQOL-54 Cognitive function	−0.309 (0.117)	0.528 (0.005)	−0.243 (0.222)	0.114 (0.578)
MSQOL-54 Health distress	−0.197 (0.326)	0.422 (0.028)	−0.306 (0.120)	0.140 (0.494)
MSQOL-54 Physical health score	−0.313 (0.127)	0.554 (0.004)	−0.141 (0.502)	−0.051 (0.814)
MSQOL-54 Mental health score	−0.232 (0.244)	0.403 (0.037)	−0.174 (0.384)	0.047 (0.820)
Positivity Scale	0.090 (0.655)	−0.005 (0.978)	−0.102 (0.613)	0.021 (0.918)

BFI: Big Five Inventory; CES-D: Center for Epidemiological Studies–Depression; MSQOL: Multiple Sclerosis Quality of Life; OSSS-3: Oslo Social Support Scale-3. Pearson correlation coefficients (r) and corresponding two-tailed p-values are presented. Statistical significance was set at $p < 0.05$.

This content has been provided by the author(s) and has not been reviewed, verified, or endorsed by European Publishing. It may not have undergone peer review. The views, opinions, and recommendations expressed are solely those of the author(s) and do not necessarily reflect the position of European Publishing. European Publishing accepts no responsibility or liability for any consequences arising from the use of, or reliance on, this content.
